# Lattice Oxygen Redox Dynamics in Zeolite‐Encapsulated CsPbBr_3_ Perovskite OER Electrocatalysts

**DOI:** 10.1002/advs.202412679

**Published:** 2025-01-09

**Authors:** Xiangrong Ren, Yiyue Zhai, Na Yang, Bolun Wang, Shengzhong (Frank) Liu

**Affiliations:** ^1^ Key Laboratory of Applied Surface and Colloid Chemistry Ministry of Education Shaanxi Key Laboratory for Advanced Energy Devices Shaanxi Engineering Lab for Advanced Energy Technology School of Materials Science and Engineering Shaanxi Normal University Xi'an 710119 P. R. China; ^2^ School of Civil and Architecture Engineering Xi'an Technological University Xi'an 710021 P. R. China; ^3^ School of Materials and Energy University of Electronic Science and Technology of China Chengdu 611731 P. R. China; ^4^ State Key Laboratory of Inorganic Synthesis and Preparative Chemistry College of Chemistry International Center of Future Science Jilin University Changchun 130012 P. R. China; ^5^ Key Laboratory of Photoelectric Conversion and Utilization of Solar Energy Dalian Institute of Chemical Physics Chinese Academy of Sciences Dalian Liaoning 116023 P. R. China; ^6^ Center of Materials Science and Optoelectronics Engineering University of Chinese Academy of Sciences Beijing 100049 P. R. China; ^7^ CNNP Optoelectronics Technology 2828 Canghai Road Lingang Shanghai 201308 China

**Keywords:** electrocatalyst, halide perovskites, OER, surface reconstruction, zeolites

## Abstract

Understanding the oxygen evolution reaction (OER) mechanism is pivotal for improving the overall efficiency of water electrolysis. Despite methylammonium lead halide perovskites (MAPbX_3_) have shown promising OER performance due to their soft‐lattice nature that allows lattice‐oxygen oxidation of active α‐PbO_2_ layer surface, the role of A‐site MA or X‐site elements in the electrochemical reconstruction and OER mechanisms has yet to be explored. Here, it is demonstrated that the OER mechanism of perovskite@zeolite composites is intrinsically dominated by the A‐site group of lead‐halide perovskites, while the type of X‐site halogen is crucial for the reconstruction kinetics of the composites. Using CsPbBr*_x_* I_3‐_ *_x_* @AlPO‐5 (*x* = 0, 1, 2, 3) as a model OER catalyst, it is found that the CsPbBr_3_@AlPO‐5 behaves oxygen‐intercalation pseudocapacitance during surface restructuring due to absence of halogen‐ion migration and phase separation in the CsPbBr_3_, achieving a larger diffusion rate of OH^−^ within the core‐shell structure. Moreover, distinct from the single‐metal‐site mechanism of MAPbBr_3_@AlPO‐5, experimental and theoretical investigations reveal that the soft lattice nature of CsPbBr_3_ triggers the oxygen‐vacancy‐site mechanism via the CsPbBr_3_/α‐PbO_2_ interface, resulting in excellent OER performance. Owing to the variety and easy tailoring of lead‐halide perovskite compositions, these findings pave a way for the development of novel perovskite@zeolite type catalysts for efficient oxygen electrocatalysis.

## Graphical abstract

The OER mechanism of halide‐perovskite@zeolite composites is intrinsically dominated by the A‐site group of perovskites, while the type of X‐site halogen is crucial for electrochemical reconstruction kinetics. Distinct from the single‐metal‐site mechanism of MAPbBr_3_@AlPO‐5, the soft‐lattice nature of CsPbBr_3_ triggers the oxygen‐vacancy‐site mechanism via the CsPbBr_3_/α‐PbO_2_ interface, outperforming the benchmark IrO_2_@NF electrocatalysts.

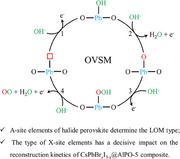

## 1 Introduction

Water electrolysis using renewable energy input has been spotlighted as an efficient and sustainable route for hydrogen fuel production.^[^ ^1^, ^2^, ^3^ ^]^ Nevertheless, the sluggish kinetics of the oxygen evolution reaction (OER) have long posed the main bottleneck for large‐scale application of water electrolyzers.^[^ ^4^, ^5^, ^6^, ^7^, ^8^, ^9^, ^10^ ^]^ The challenge remains that inexpensive and earth‐abundant OER electrocatalysts are hard to fulfill the needs of high‐efficiency and long‐term durability concurrently.^[^ ^11^, ^12^, ^13^, ^14^, ^15^, ^16^, ^17^, ^18^ ^]^ Recently, substantial progress toward OER mechanisms was made in perovskite oxides,^[^ ^19^ ^]^ namely the lattice‐oxygen oxidation mechanism (LOM), which proceeds through lattice‐oxygen redox with oxygen bands around the Fermi level (*E*_F_) and breaks the scaling relationship in mononuclear adsorbate evolution mechanism (AEM), achieving higher intrinsic OER activity.^[^ ^20^, ^21^, ^22^ ^]^ To date, three kinds of LOM pathways with different active centers have been proposed, that is, oxygen‐vacancy‐site mechanism (OVSM), single‐metal‐site mechanism (SMSM), and dual‐metal site mechanism (DMSM), respectively.^[^ ^10^, ^20^ ^]^

Generally, manipulating the electronic structures of the transition metal (TM) oxides/oxyhydroxides, such as the upshift of the O 2p band and the downshift of the lower Hubbard band (LHB), have been demonstrated as effective ways to trigger the LOM.^[^ ^23^ ^]^ However, the rigid characteristic of metal‐oxygen bonds still leads to a higher free‐energy change during the OER.^[^ ^20^, ^24^ ^]^ Inspired by the soft‐lattice nature of halide perovskites, we first demonstrated the crucial role of methylammonium lead halide perovskites (MAPbX_3_) in producing oxygen vacancies (V_O_s) for MAPbX_3_@AlPO‐5 composite, where aluminophosphates (AlPO‐5) with well‐defined channels are ideal host matrices owing to their low cost, large specific surface area, easy processability, and high thermal stability, which substantially reduces the adsorption free‐energy of oxygenated intermediates, activating the SMSM during water oxidation.^[^ ^25^ ^]^ Given the easy processability and variety of halide perovskite compositions,^[^ ^26^, ^27^, ^28^, ^29^, ^30^ ^]^ the question then arises: first, whether changes in the A‐site elements affect the LOM mechanism of the composites is still unanswered; second, MAPbX_3_@AlPO‐5 undergoes surface restructuring preceding the OER, what roles do the A‐site or X‐site elements of lead halide perovskites play in the electrochemical reconstruction of perovskite@zeolite composites?

Here, we investigate the chemical origin of the LOM in perovskite@zeolite composites using CsPbBr*_x_* I_3‐_ *_x_* @AlPO‐5 (*x* = 0, 1, 2, 3) as model materials, disclosing the impact of changing the A‐site or X‐site elements of lead‐halide perovskites on the surface reconstruction and LOM mechanisms of composites. The prepared CsPbBr*_x_* I_3‐_ *_x_* @AlPO‐5 exhibits excellent water stability due to the effective passivation of AlPO‐5 zeolite on the CsPbBr*_x_* I_3‐_ *_x_* defects via electrostatic and hydrogen‐bond interactions. Similar to the surface restructuring of MAPbBr*_x_* I_3‐_ *_x_* @AlPO‐5, CsPbBr*_x_* I_3‐_ *_x_* @AlPO‐5 undergoes electrochemical reconstruction to form a surface‐active layer of α‐PbO_2_ during anodic water oxidation, suggesting that the surface reconstruction of the perovskite@zeolite composite is independent of the type of A‐site element. In contrast, changes in the halogen at the X‐site have a decisive impact on the reconstruction kinetics of the composite. The CsPbBr_3_@AlPO‐5 features pseudocapacitive behavior during surface restructuring owing to the absence of halogen ion migration and phase segregation in perovskite CsPbBr_3_, enabling a higher diffusion rate of OH^−^. More importantly, the A‐site elements of halide perovskites determine the LOM mechanism of the surface α‐PbO_2_ active layer, with the CsPbBr_3_/α‐PbO_2_ interface triggering the OVSM pathway during water oxidation, which differs from the SMSM of MAPbBr_3_@AlPO‐5. Following the OVSM, CsPbBr_3_@AlPO‐5 ultimately achieves a high current density of 100 mA cm^−2^ at an overpotential of 357 mV, outperforming the benchmark IrO_2_@NF electrode.^[^ ^31^, ^32^, ^33^, ^34^, ^35^, ^36^ ^]^

## 2 Results and Discussion

### 2.1 Structural Characterizations

**Figure 1:**
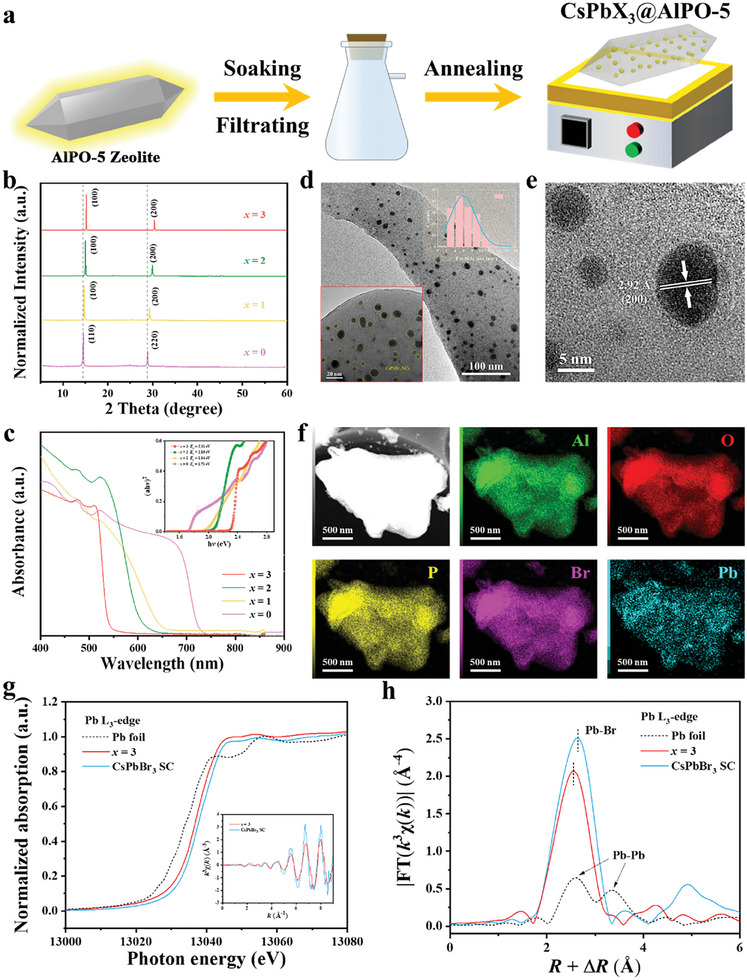
a) Schematic of the preparation process of CsPbX_3_@AlPO‐5.^[^ ^37^ ^]^ b) GIXRD patterns and c) UV–vis absorption spectra of CsPbBr*_x_* I_3‐_ *_x_* @AlPO‐5 (*x* = 0, 1, 2, 3). Inset of (c): Tauc plots. d) TEM image, e) HRTEM image, f) HAADF‐STEM image and elemental mappings of CsPbBr_3_@AlPO‐5. Right‐top and bottom‐left insets of d) give the particle‐size distribution of CsPbBr_3_ NCs and a zoomed region of the TEM image, respectively. g) Normalized Pb L_3_‐edge XANES spectra and h) Fourier‐transformed *k* ^3^‐weighted EXAFS spectra of CsPbBr_3_@zeolite and CsPbBr_3_ single crystal (SC) with Pb foil as a reference. Inset of (g): Pb L_3_‐edge EXAFS oscillation functions *k* ^3^χ(*k*).

The CsPbX_3_@AlPO‐5 composite was prepared by impregnation and annealing method, as illustrated in **Figure** **1**a.^[^ ^37^ ^]^ Grazing‐incidence X‐ray diffraction (GIXRD) patterns (Figure 1b) confirm the pure cubic CsPbBr_3_ phase of CsPbBr_3_@AlPO‐5 with (100) facet as the dominating facet,^[^ ^38^ ^]^ where the diffraction peaks at 15.19° and 30.64° are consistent with the (100) and (200) crystal planes, respectively. In particular, the main diffraction peak of CsPbBr*_x_* I_3‐_ *_x_* @AlPO‐5 (*x* = 0, 1, 2, 3) shifts to larger angles with increasing bromine content, and the preferred growth of the crystal face changes from the (110) facet of CsPbI_3_@AlPO‐5 to the (100) facet of CsPbBr_3_@AlPO‐5. Note that the diffraction peaks of AlPO‐5 zeolites were observed on CsPbBr*_x_* I_3‐_ *_x_* @AlPO‐5 composites (Figure S1, Supporting Information), indicating that the AlPO‐5 matrix is not destroyed after the incorporation with CsPbBr*_x_* I_3‐_ *_x_* (see Figure S2, Supporting Information for more details of AlPO‐5).

UV–vis absorption spectra (Figure 1c) reflect that the optical band gap of CsPbBr*_x_* I_3‐_ *_x_* (*x* = 0, 1, 2, 3) becomes wide as the Br content increases, which agrees with the previous report of halide‐perovskite polycrystals, demonstrating a clear halogen‐dependent band structure of CsPbBr*_x_* I_3‐_ *_x_* perovskites embedded in the AlPO‐5 matrix.^[^ ^39^ ^]^ In addition, for each composition, a noticeable shift of the band edge to higher energies in contrast to corresponding polycrystalline powders is a signature of quantum confinement of excitons within the CsPbBr*_x_* I_3‐_ *_x_* in composites.^[^ ^26^ ^]^ To investigate the particle size of CsPbBr*_x_* I_3‐_ *_x_* in detail, the morphology of CsPbBr*_x_* I_3‐_ *_x_* @AlPO‐5 composites was characterized by transmission electron microscopy (TEM). Taking CsPbBr_3_@AlPO‐5 as an example, Figure 1d manifests that the uniform and monodispersed CsPbBr_3_ nanocrystals (NCs) are distributed within the zeolite matrix with an average size of 3.5 nm. The high‐resolution TEM (HRTEM) image in Figure 1e exhibits a well‐resolved lattice spacing of 2.92 Å ascribed to the (200) plane of the cubic CsPbBr_3_ phase.^[^ ^38^ ^]^ In light of the CsPbBr_3_ NCs being larger than the intrinsic micropores of AlPO‐5 (7.3 × 7.3 Å), we believe the CsPbBr_3_ NCs were embedded in the interrupted spaces of AlPO‐5 matrix, which has also been reported in carbon dots@zeolite composites.^[^ ^40^, ^41^, ^42^ ^]^ Besides, the high‐angle annular dark‐field scanning TEM (HAADF‐STEM) image (Figure 1f) confirms that the CsPbBr_3_ NCs generated in zeolite crystals are non‐agglomerated. Accordingly, energy‐dispersive X‐ray spectroscopy (EDS) element mapping exhibits that the elements Al, O, P, Br, and Pb are homogeneously distributed in the CsPbBr_3_@AlPO‐5, and the atomic ratio of Pb/Br is 1:2.86, which is in accordance with the results of the inductively coupled plasma mass spectrometry (ICP‐MS) (Table S1, Supporting Information).

Considering there are massive dangling functional groups, such as Al‐OH and P‐OH, at the interrupted nanospaces of AlPO‐5 matrices, complex hydrogen‐bonding interactions could be formed between the zeolite frameworks and halide anions of CsPbBr*_x_* I_3‐_ *_x_* NCs, as confirmed by the Fourier transform infrared (FT‐IR) spectra in Figure S3 (Supporting Information). In comparison to the neat AlPO‐5 template, the broad peak ascribed to the O‐H stretch of CsPbBr*_x_* I_3‐_ *_x_* @AlPO‐5 (*x* = 0, 1, 2, 3) delivers an evident shift to lower wavenumber, which indicates the hydroxy groups situated at interruptions in AlPO‐5 frameworks strongly interact with halide anions via hydrogen‐bond interactions, significantly enhancing the cohesion of the CsPbBr*_x_* I_3‐_ *_x_* NCs with the zeolite matrices.^[^ ^43^ ^]^ In particular, the proton conductivity of the CsPbBr_3_@AlPO‐5 is two orders of magnitude higher than that of the pristine AlPO‐5 matrix (Figure S4 and Table S2, Supporting Information); the presence of hydrogen bonds substantially offers more channels for proton transfer, resulting in high proton conductivity.

We further investigated the local coordination environment of the CsPbBr_3_@zeolite and CsPbBr_3_ single crystal (SC) using X‐ray absorption near‐edge structure (XANES) measurements and extended X‐ray absorption fine structure (EXAFS) spectroscopy. As observed in the normalized lead L_3_‐edge XANES spectra (Figure 1g), the absorption edge position of the CsPbBr_3_@zeolite approaches the position of Pb^2+^ in CsPbBr_3_ SC, verifying a Pb^2+^ valence state in the CsPbBr_3_@zeolite. The lead L_3_‐edge *k* ^3^χ(*k*) oscillation curve of the CsPbBr_3_@zeolite reflects subtle reductions in oscillation amplitudes (inset in Figure 1g), implying a coordination environment change of lead atoms compared with the CsPbBr_3_ SC. Moreover, Fourier transform of the lead L_3_‐edge EXAFS spectrum (Figure 1h) discloses that the dominant peak of the CsPbBr_3_@zeolite at 2.93 Å is assigned to the Pb‐Br bond, which is shorter than that of CsPbBr_3_ SC (2.98 Å), indicating the zeolite matrix provides electronic confinement for CsPbBr_3_ nanocrystals (Figure S5 and Table S3, Supporting Information).

X‐ray photoelectron spectroscopy (XPS) analysis was also performed to disclose the host‐guest interactions between the zeolitic matrices and perovskite NCs. Assisted by the electronic confinement from the AlPO‐5 host, the orbital energy of the perovskite NCs guest increases, favoring a stronger coulombic interaction between the guest perovskite NCs and the host AlPO‐5.^[^ ^44^ ^]^ In high‐resolution O 1*s* spectra (Figure S6a, Supporting Information), the dominant peak of the CsPbBr_3_@AlPO‐5 displays a left shift of 0.24 eV to higher binding energy compared to neat AlPO‐5 zeolite, while the peak position in P 2*p* and Al 2*p* spectra shifts to higher binding energies by 0.16 and 0.23 eV, respectively, (Figure S6b,c and Table S4, Supporting Information), suggesting that the electronic interactions with CsPbBr_3_ NCs are derived from the TO_4_ tetrahedra (T = Al and P) in the zeolite frameworks (similar results were observed on CsPbBr*_x_* I_3‐_ *_x_* @AlPO‐5 (*x* = 0, 1, 2), Figure S7, Supporting Information).^[^ ^41^ ^]^

On the basis of the above observations, we conclude that AlPO‐5 matrices not only provide quantum and electronic confinement for CsPbBr*_x_* I_3‐_ *_x_* NCs but also passivate the perovskite defects through coulombic and hydrogen‐bond interactions, resulting in the superior water stability of CsPbBr*_x_* I_3‐_ *_x_* @AlPO‐5.

### 2.2 Electrocatalytic OER Performance

**Figure 2:**
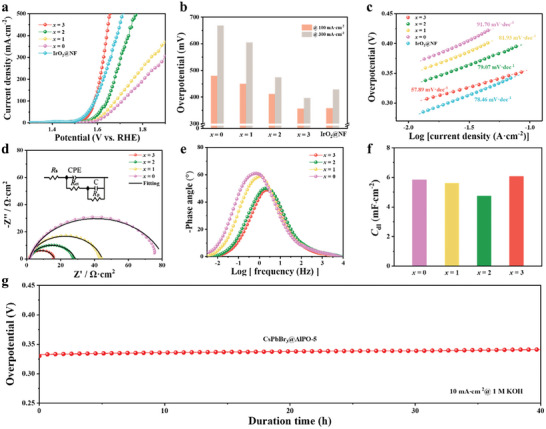
a) Polarization curves, b) overpotentials at 100 and 300 mA·cm^−2^, c) Tafel slopes, d) Nyquist plots at 1.48 V_RHE_, e) Bode plots, and f) *C*_dl_ of CsPbBr*_x_* I_3‐_ *_x_* @AlPO‐5 (*x* = 0, 1, 2, 3). g) Chronopotentiometry of CsPbBr_3_@AlPO‐5 at 10 mA·cm^−2^.

The OER activities of CsPbBr*_x_* I_3‐_ *_x_* @AlPO‐5 (*x* = 0, 1, 2, 3) were evaluated in O_2_‐saturated 1 m KOH using a three‐electrode set‐up. As shown in **Figure** **2**a,b, CsPbBr_3_@AlPO‐5 delivers an overpotential of 357 mV to achieve a current density of 100 mA·cm^−2^, smaller than that of the CsPbIBr_2_@AlPO‐5 (412 mV), CsPbI_2_Br@AlPO‐5 (450 mV) and CsPbI_3_@AlPO‐5 (480 mV). Moreover, CsPbBr_3_@AlPO‐5 features the lowest Tafel slope of 57.89 mV·dec^−1^ (Figure 2c), compared with CsPbIBr_2_@AlPO‐5 (79.07 mV·dec^−1^), CsPbI_2_Br@AlPO‐5 (81.93 mV·dec^−1^), CsPbI_3_@AlPO‐5 (91.70 mV·dec^−1^), and commercial IrO_2_@NF (78.46 mV·dec^−1^), suggesting its fast reaction kinetics with the generation of *OOH being the rate‐determining step (RDS) during the OER.^[^ ^45^, ^46^ ^]^ Using electrochemical impedance spectroscopy (EIS), the smallest polarization resistance of 14.96 Ω·cm^2^ was determined in CsPbBr_3_@AlPO‐5, where the resistance increases with decreasing Br content (Figure 2d and Table S5, Supporting Information), demonstrating its enhanced charge transfer ability. Also, Figure 2e shows the smallest phase angle of CsPbBr_3_@AlPO‐5 among the tested composites at the same potential, reflecting that more electrons are involved in oxygen electrocatalysis of CsPbBr_3_@AlPO‐5, in agreement with the Tafel analysis (see Figure S8, Supporting Information for the comparison of OER performance among various perovskite@zeolite composites).^[^ ^47^ ^]^

The electrochemical surface areas (ECSA) were estimated from the calculation of double‐layer capacitance (*C*_dl_) at the non‐Faradic region, as displayed in Figure 2f. The highest *C*_dl_ value of 6.06 mF·cm^−2^ was discovered in CsPbBr_3_@AlPO‐5, which indicates that the enhanced OER activity benefits from the increased number of active sites. The stability of the CsPbBr_3_@AlPO‐5 was tested under a constant current continuously for 40 h (Figure 2g), where CsPbBr_3_@AlPO‐5 exhibits remarkable stability with stable potential during water oxidation.

### 2.3 Surface Oxidation and Reconstruction

**Figure 3:**
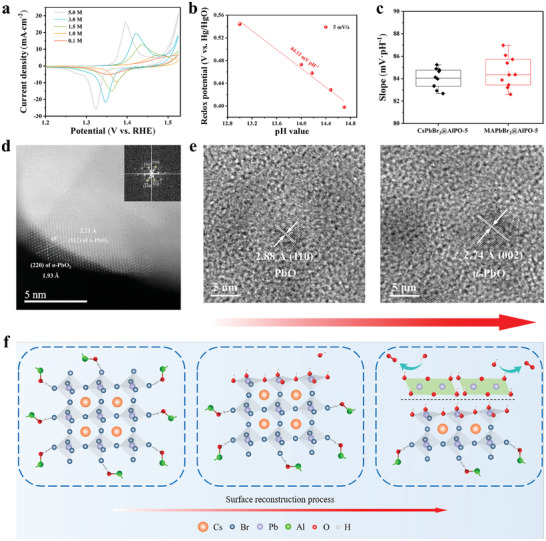
a) CV curves and b) Pourbaix diagrams of CsPbBr_3_@AlPO‐5 in O_2_‐saturated 0.1 to 5.0 m KOH. Scan rate: 5 mV s^−1^. c) The comparison of the slope of CsPbBr_3_@AlPO‐5 and MAPbBr_3_@AlPO‐5 at various scan rates. d) iDPC‐STEM image of composites after water oxidation. Inset: the related FFT pattern. e) Ex situ HRTEM of CsPbBr_3_@AlPO‐5 with different oxidation times. f) Schematic of the restructuring process of CsPbBr_3_@AlPO‐5.

Given the restructuring of MAPbBr*_x_* I_3‐_ *_x_* @AlPO‐5 in our previous reports,^[^ ^25^ ^]^ we started with cyclic voltammetry (CV) measurements at various pH levels to investigate the surface evolution of CsPbBr*_x_* I_3‐_ *_x_* @AlPO‐5. As displayed in **Figure** **3**a, oxidation peaks were observed on CsPbBr_3_@AlPO‐5 at varying pH levels, which signifies a chemical reconstruction process preceding the OER. Moreover, the redox potentials of CsPbBr_3_@AlPO‐5 depend linearly on the pH level with the slope of 84.12 mV·pH^−1^, which suggests its reconstruction process involving an OH^−^ (H^+^)/e stoichiometry of 3/2 (Figure 3b).^[^ ^48^ ^]^ We subsequently compared the slope of CsPbBr_3_@AlPO‐5 and MAPbBr_3_@AlPO‐5 composites at different scan rates and found that their slope distributions are highly similar (Figure 3c), which discloses that the type of A‐site cations in halide perovskites does not correlate with the three‐proton and two‐electron process of perovskite@zeolite composites during electrochemical reconstruction.

With the assistance of integrated differential phase contrast STEM (iDPC‐STEM), we further investigated the microscopic structural changes of CsPbBr_3_@zeolite that had undergone 3‐h oxidation, as shown in Figure 3d. Obviously, iDPC‐STEM observation manifests lattice stripes corresponding to the (112) and (220) facets of *α*‐PbO_2_, with an angle of 59° between them. Given that the reconstruction of CsPbBr_3_@AlPO‐5 occurs in an alkaline electrolyte, a hydrated layer will initially form on CsPbBr_3_@AlPO‐5 surface solely driven by thermodynamics when the potential has not reached ≈1.48 V_RHE_ (in 1 m KOH),^[^ ^25^ ^]^ where lead ions remain as Pb^2+^. To test this hypothesis experimentally, ex situ HRTEM characterization was conducted, from which CsPbBr_3_@AlPO‐5 was either soaked in 1 m KOH for 1 h or pre‐oxidized for 3 h. As shown in Figure 3e, the left HRTEM image reveals clear lattice fringes ascribed to the (110) plane of PbO, with an interplanar spacing of 2.88 Å; the right image indicates the formation of α‐PbO_2_, corresponding to the (002) facet. This demonstrates that the CsPbBr_3_ in the composite indeed requires a potential of 1.48 V_RHE_ to undergo the Pb^2+^/Pb^4+^ oxidation, in line with the CV results in Figure 3a (Figure S9, Supporting Information also verifies that the valence state of Pb remains unchanged when CsPbBr_3_@AlPO‐5 was soaked in 1 m KOH for 3 h).

As mentioned previously, the predominant plane of perovskite CsPbBr_3_ in CsPbBr_3_@AlPO‐5 is the (100) facet that belongs to the {100} plane family with identical crystal structures in the cubic CsPbBr_3_ phase. Based on the above observations, we infer that during the electrochemical reconstruction process, *α*‐PbO_2_ grows with an orientation relationship of {100}CsPbBr_3_//{100}*α*‐PbO_2_, leading to the preferential exposure of the {100} family of crystal faces of *α*‐PbO_2_. Furthermore, ICP‐MS results in Table S1 (Supporting Information) reveals a molar ratio of 1.06:1 for Pb/Br in CsPbBr_3_@AlPO‐5 after oxygen electrocatalysis, which indicates that nearly two‐thirds of the Br^−^ in CsPbBr_3_@AlPO‐5 is replaced by OH^−^ during the electrochemical process, as illustrated in Figure 3f.

**Figure 4:**
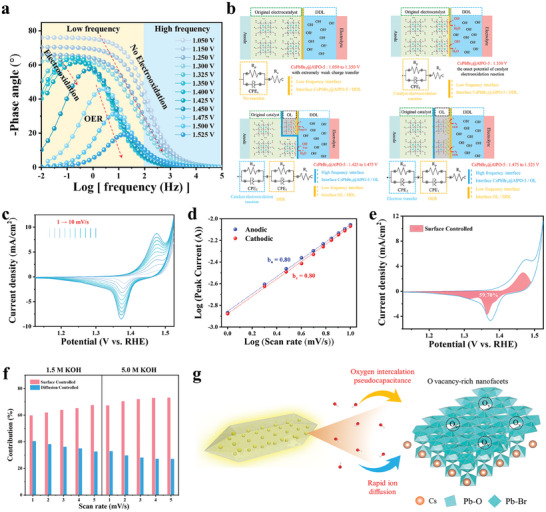
a) Bode plots and b) Schematic of an equivalent circuit model of CsPbBr_3_@AlPO‐5 at 1.050–1.525 V_RHE_. c) CV curves from 1 to 10 mV s^−1^, d) the relationship between peak currents and sweet rates, and e) pseudocapacitive contributions for CsPbBr_3_@AlPO‐5 at 5 mV s^−1^. f) Pseudocapacitive contribution distribution of CsPbBr_3_@AlPO‐5 from 1 to 5 mV s^−1^. g) Schematic of the pseudocapacitance effect for CsPbBr_3_@AlPO‐5 during water oxidation.

To further track the dynamic changes of Pb sites of CsPbBr_3_@AlPO‐5 during water oxidation, *operando* EIS tests were conducted to gain deeper insights into reaction kinetics at electrode‐electrolyte interfaces. Based on the Nyquist plots (Figure S10, Supporting Information) and the Bode plots (**Figure** **4**a) of CsPbBr_3_@AlPO‐5 composite, two different equivalent circuit models of *R_s_* (*Q*_1_ *R*_p_) and *R*_s_(*Q*_1_ *R*_p_)(*Q*_2_ *R*_ct_) represent the interfacial kinetic processes before or after electrooxidation reaction, respectively, where *R*_s_ stands for the solution resistance while *R*_p_ and *R*_ct_ denote the resistances for interfacial reactions in the low‐frequency and high‐frequency regions, respectively. Combining previous CV results in 1 m KOH (Figure 3a), it can be seen that before 1.35 V_RHE_, CsPbBr_3_@AlPO‐5 did not undergo surface reconstruction due to the large electrochemical polarization resistance. During this stage, only the replacement of halide anions on the CsPbBr_3_ NC surface by OH^−^ ions or H_2_O molecules was achieved under thermodynamic driving, forming a [Pb_2_(OH)_6_∙3H_2_O]^2−^ hydrated layer. When the electrode potential rises to 1.35 V_RHE_, the electrochemical oxidation reaction of CsPbBr_3_@AlPO‐5 initiates as *R*_p_ continues to decrease, which corresponds to the low‐frequency interfacial reaction between CsPbBr_3_@AlPO‐5 and the diffuse double layer (DDL). However, it should be noted that the oxidation current at this stage is relatively small, and the *α*‐PbO_2_ phase has not yet formed. As the potential gradually increases, from 1.425 to 1.475 V_RHE_, an oxide layer (OL) begins to appear on the surface of CsPbBr_3_@AlPO‐5, corresponding to the high‐frequency interfacial reaction between CsPbBr_3_@AlPO‐5 and the OL. Note that although the new interface between CsPbBr_3_ and *α*‐PbO_2_ has formed, the effective electron transfer at the CsPbBr_3_/α‐PbO_2_ interface (as will be supported by subsequent theoretical calculations) results in a small interfacial resistance *R*_ct_, making it difficult for the capacitive arc in the high‐frequency region to be displayed in the impedance spectrum (Table S6, Supporting Information).

After 1.48 V_RHE_, the OER takes place at the low‐frequency interface between surface *α*‐PbO_2_ (OL) and the DDL with a significant reduction in the phase angle (Figure 4a), in line with the results of the Faradaic efficiency (FE) analysis displayed in Figure S11 (Supporting Information) (the FE of CsPbBr_3_@AlPO‐5 reaches almost 100% without other side reactions). Accordingly, from 1.050 to 1.525 V_RHE_, the evolution process of the electrode‐electrolyte interface reaction of CsPbBr_3_@AlPO‐5 is depicted in Figure 4b.

To scrutinize the electrochemical behavior of CsPbBr*_x_* I_3‐_ *_x_* @AlPO‐5 (*x* = 0, 1, 2, 3) during surface reconstruction, CV measurements were conducted from 1 to 10 mV s^−1^. As seen in Figure 4c, CsPbBr_3_@AlPO‐5 shows an oxidation peak of Pb^2+^/Pb^4+^ at ca. 1.48 V_RHE_, verifying the generation of the α‐PbO_2_ before water oxidation. The *b* values for the anodic and cathodic peaks of CsPbBr_3_@AlPO‐5 were both found to be 0.80 through the logarithmic relationship between peak current and sweet rate (Figure 4d), which suggests that the kinetics are predominantly governed by surface‐controlled behavior from 1 to 10 mV s^−1^, while also demonstrating the best electrochemical reversibility among CsPbBr*_x_* I_3‐_ *_x_* @AlPO‐5 composites (see detailed discussion of CsPbBr*_x_* I_3‐_ *_x_* @AlPO‐5 (*x* = 0, 1, 2) in Figures S12–S14 (Supporting Information). The detailed contribution of surface‐controlled behavior in Figure 4e displays that 59.70% of the total current arises from pseudocapacitive contributions at 5 mV s^−1^, which will benefit its electrocatalytic OER activity. Importantly, we discovered that the proportion of pseudocapacitive contributions increases with bromine content in CsPbBr*_x_* I_3‐_ *_x_* @AlPO‐5 composites, which discloses that the type of the halogen in CsPbBr*_x_* I_3‐_ *_x_* has important implications for the reconstruction kinetics of the composites (where CsPbBr_3_@AlPO‐5 possesses larger diffusion coefficient of OH^−^ ions in comparison with MAPbBr_3_@AlPO‐5, Table S7, Supporting Information).

Given the strong pH dependence of the location of Pb^2+^/Pb^4+^ redox peak in Figure 3a, the mechanism of pseudocapacitance of CsPbBr_3_@AlPO‐5 belongs to an anion‐based intercalation pseudocapacitance, which uses the OH^−^ as the intercalating ion.^[^ ^49^, ^50^ ^]^ We further evaluated the electrochemical behavior of CsPbBr_3_@AlPO‐5 in potassium‐based electrolytes with elevating pH levels. As shown in Figure 4f, pseudocapacitive contribution of CsPbBr_3_@AlPO‐5 increases dramatically with the pH level, where the OER activity was found to present a similar trend when the pH gradient enlarges (Figure 3a). Based on the above findings, we conclude that the soft‐lattice nature of CsPbBr_3_ enables easier electrochemical intercalation of OH^−^ ions in V_O_s of reconstructed CsPbBr_3_@AlPO‐5 surface at higher pH conditions, featuring an enhanced pseudocapacitance contributions controlled by surface reactions that will boost the formation of *OH during water oxidation, as depicted in Figure 4g. In addition, the pH‐dependent OER activity of CsPbBr_3_@AlPO‐5 may respond directly to the LOM, which will be discussed later.

### 2.4 Chemical Origin of the LOM

**Figure 5:**
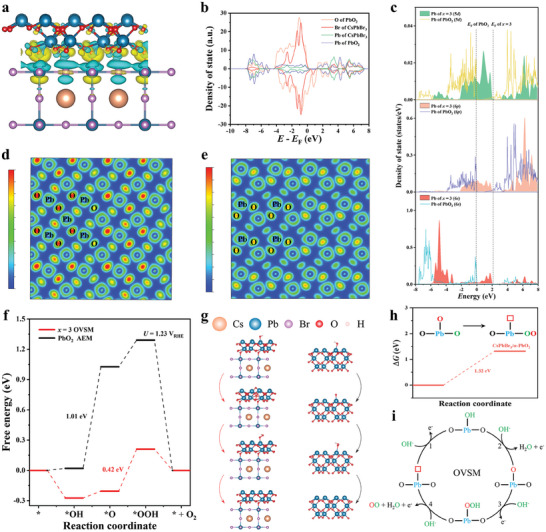
a) Charge density difference of CsPbBr_3_/α‐PbO_2_. Yellow and blue colors represent charge accumulation and loss, separately. b) Density of states (DOS) of CsPbBr_3_/α‐PbO_2_. c) Projected DOS of Pb 5*d*, 6*s*, and 6*p* orbitals of surface α‐PbO_2_ in CsPbBr_3_/α‐PbO_2_ and pristine α‐PbO_2_. The *E*_F_ of pristine α‐PbO_2_ is set to zero, with the abscissa versus the vacuum level. d,e) ELF contour map of CsPbBr_3_/α‐PbO_2_ d) and pure α‐PbO_2_ e), ranging from 0 to 0.6. f,g) The OER free‐energy f) and adsorption configurations g) on CsPbBr_3_/α‐PbO_2_ and pristine α‐PbO_2_. Dark blue, red, purple, and orange balls signify lead, oxygen, bromine, and cesium atoms, separately. h) Free energies of SMSM steps on CsPbBr_3_/α‐PbO_2_. i) Proposed OVSM of CsPbBr_3_/α‐PbO_2_. The chemically inert lattice oxygen, active lattice oxygen involving the OER, and the oxygen from the electrolyte are represented in black, red, and green colors, respectively.

To uncover the chemical origin of labile nature in reconstructed α‐PbO_2_ and determine the OER mechanism of CsPbBr_3_@AlPO‐5, density functional theory (DFT) calculations were conducted, where a model of CsPbBr_3_/α‐PbO_2_ was constructed according to experimental observations (the reconstructed CsPbBr_3_/α‐PbO_2_ and zeolite matrices are well retained after OER stability test, details in Figures S15 and S16, Supporting Information). As displayed in **Figure** **5**a, the charge density difference plot reveals effective charge transfer at the CsPbBr_3_/α‐PbO_2_ interface, which well explains why the interfacial resistance *R*_ct_ of CsPbBr_3_/α‐PbO_2_ is minimal (Figure 4b). Based on the density of state (DOS) analysis (Figure 5b), the peak overlap in low‐energy region (−8 to −5 eV) reflects orbital hybridization at the CsPbBr_3_/α‐PbO_2_ interface, in which stable Pb‐Br, Pb‐O, and Br‐O bonds were formed. Furthermore, the *E*_F_ of CsPbBr_3_/α‐PbO_2_ significantly shifts upward (−1.68 eV, versus the vacuum level) compared to pure α‐PbO_2_ (−3.80 eV), where the electronic states of Pb‐5*d*, Pb‐6*s* and Pb‐6*p* orbitals near the *E*_F_ could be found (Figure 5c; Figure S17, Supporting Information), which is beneficial for extracting electrons at surface lead centers of CsPbBr_3_/α‐PbO_2_ when bonding with oxygenated intermediates. Also, for CsPbBr_3_/α‐PbO_2_, the O 2p band of surface α‐PbO_2_ moves up compared with pure α‐PbO_2_ (Figure S18, Supporting Information); the existence of partially labile electrons near the *E*_F_ will facilitate the formation of ligand oxygen holes.^[^ ^51^ ^]^

To further clarify the differences in the electronic structure of surface Pb centers between CsPbBr_3_/α‐PbO_2_ and pure α‐PbO_2_, the electron localization function (ELF) was subsequently calculated. As shown in Figure 5d,e, where the 2D slice of ELF is derived from the cross‐sectional plane marked in 3D ELF maps by the red lines (Figure S19, Supporting Information), the ELF values present a significant increase of the electron density around the surface O bound to the Pb active‐center in CsPbBr_3_/α‐PbO_2_, demonstrating that the geometric changes induced by the CsPbBr_3_/α‐PbO_2_ interface contribute to the alteration of the electronic structure of the surface α‐PbO_2_. In addition, it was demonstrated that the formation of a single V_O_ at bridging oxygen is thermodynamically favorable in the surface α‐PbO_2_ of CsPbBr_3_/α‐PbO_2_ (Figures S20, S21, Supporting Information), which suggests that the lattice oxygens at the bridging sites can participate in the OER process, disclosing the soft lattice nature of CsPbBr_3_ indeed facilitate the formation of V_O_ on the α‐PbO_2_ surface.^[^ ^19^, ^45^ ^]^ Accordingly, the computed free energy of water oxidation in Figure 5f reflects the preferential OVSM pathway of CsPbBr_3_/α‐PbO_2_ with a free‐energy change of 0.42 eV for the potential‐determining step (PDS), in which the formation of *OOH serves as the PDS, consistent with the previous Tafel results (where the adsorption configurations of CsPbBr_3_/α‐PbO_2_ are described in Figure 5g). For comparison, pristine α‐PbO_2_ only follows the mononuclear AEM and the formation of *O acts as the PDS, together with a higher free‐energy change of 1.01 eV.^[^ ^52^ ^]^ From these results we posit that the activated lattice oxygen at the bridge site in surface α‐PbO_2_ substantially reduces the free‐energy change when oxygen‐containing intermediate *O forms due to the existence of CsPbBr_3_/α‐PbO_2_ interface, highlighting the crucial role of perovskite CsPbBr_3_ featuring soft lattice in steering electronic structure of α‐PbO_2_ surfaces (Figure S22, Supporting Information further verifies that when changing the ratio of the precursor solution, the composites display poor OER performance compared with CsPbBr_3_@AlPO‐5 composite).

Additionally, Figure 5h displays that the direct coupling of oxygenated intermediate *O with surface bridge‐oxygen in CsPbBr_3_/α‐PbO_2_ is energetically unfavorable via the SMSM pathway, with a free energy change of 1.32 eV, which is significantly higher than the PDS of the OVSM pathway (0.42 eV), uncovering the preferential OVSM pathway for CsPbBr_3_@AlPO‐5 during water oxidation (Figure 5i and Table S8, Supporting Information).^[^ ^53^, ^54^, ^55^, ^56^ ^]^

## 3 Conclusion

In summary, we have demonstrated the decisive role of A‐site elements of halide perovskites in determining the LOM mechanism of the α‐PbO_2_ active layer. With the aid of electronic and spatial confinements, the AlPO‐5 matrix effectively passivates the defects in CsPbBr*_x_* I_3‐_ *_x_* NCs through Coulombic and hydrogen bonding interactions, achieving remarkable stability in water. Also, an active α‐PbO_2_ layer forms on the surface of CsPbBr*_x_* I_3‐_ *_x_* @AlPO‐5 preceding oxygen electrocatalysis. Importantly, experimental and DFT calculations uncover that the soft‐lattice nature of CsPbBr_3_ activates the OVSM pathway through the CsPbBr_3_/α‐PbO_2_ interface, which substantially decreases the free‐energy change during the OER process, distinct from the SMSM observed in MAPbBr_3_@AlPO‐5. In addition, the type of X‐site elements is critical for the reconstruction kinetics and the absence of halide ion migration and phase segregation in CsPbBr_3_ enhances the diffusion rate of OH^−^, behaving as oxygen‐intercalation pseudocapacitance that promotes the OER kinetics at surface lattice‐oxygen sites and ultimately results in the outstanding intrinsic activity of CsPbBr_3_@AlPO‐5. Our results shed light on the key role of A‐site elements of metal‐halide perovskites in the OER mechanism of perovskite@zeolite composites, highlighting the importance of rational compositional design for engineering lead‐halide perovskites toward efficient and robust oxygen electrocatalysis.

## Conflict of Interest

The authors declare no conflict of interest.

## Supporting information



Supporting Information

## Data Availability

The data that support the findings of this study are available from the corresponding author upon reasonable request.
