# Supplementary material for: Lattice Oxygen Redox Dynamics in Zeolite‐Encapsulated CsPbBr3 Perovskite OER Electrocatalysts
Source: Adv Sci (Weinh). 2025 Jan 9;12(8):2412679. doi: 10.1002/advs.202412679 (PMC11848567; doi:10.1002/advs.202412679)
Supplement: Supplementary file 1 — Supporting Information [file ADVS-12-2412679-s001.docx]

Supporting Information

**Lattice Oxygen Redox Dynamics in Zeolite-Encapsulated CsPbBr_3_ Perovskite OER Electrocatalysts**

*Xiangrong Ren****^+^****, Yiyue Zhai****^+^****, Na Yang*, Bolun Wang*, Shengzhong (Frank) Liu**

Dr. X. R. Ren, Dr. Y. Y. Zhai, Prof. S. Z. Liu

Key Laboratory of Applied Surface and Colloid Chemistry, Ministry of Education, Shaanxi Key Laboratory for Advanced Energy Devices, Shaanxi Engineering Lab for Advanced Energy Technology, School of Materials Science and Engineering, Shaanxi Normal University, Xi’an, 710119, P. R. China

E-mail: liusz@snnu.edu.cn

Dr. B. L. Wang

State Key Laboratory of Inorganic Synthesis and Preparative Chemistry, College of Chemistry, International Center of Future Science, Jilin University, Changchun, 130012, P. R. China

E-mail: wangbolun@jlu.edu.cn

Dr. N. Yang

School of Materials and Energy, University of Electronic Science and Technology of China, Chengdu, 611731, P. R. China

E-mail: yna@uestc.edu.cn

Prof. S. Z. Liu

Key Laboratory of Photoelectric Conversion and Utilization of Solar Energy, Dalian Institute of Chemical Physics, Chinese Academy of Sciences, Dalian, 116023, Liaoning, P. R. China; Center of Materials Science and Optoelectronics Engineering, University of Chinese Academy of Sciences, Beijing, 100049, P. R. China.

E-mail: [szliu@dicp.ac.cn](mailto:szliu@dicp.ac.cn)

Dr. Y. Y. Zhai

School of Civil and Architecture Engineering, Xi’an Technological University, Xi’an 710021, P. R. China.

**Experimental Section**

**Materials**

All chemicals were used as received. Aluminum isopropoxide (≥99.5%) was supplied by Tianjin Fuchen Chemical Reagents Factory. Triethylamine (99%) was purchased from Tianjin Fuyu Fine Chemical Co. Ltd. H_3_PO_4_ (85% weight in water) and hydrogen fluoride (40% weight in water) were obtained from Beijing Chemical Reagent Factory. Lead iodide (PbI_2_, >99.99%), lead bromide (PbBr_2_, >99.99%), cesium iodide (CsI, ≥99.99%), cesium bromide (CsBr, ≥99.99%) were purchased from Xi’an Polymer Light Technology Corp. Dimethylsulfoxide (DMSO, ≥99.9%) was acquired from Guangdong Guanghua Sci-Tech Co. Ltd. Acetone (AR, 99.5%), ethanol (AR, 99.5%), TEG, and potassium hydroxide (KOH, AR) were purchased from Sinopharm Reagent Chemical Co. Ltd. Lead dioxide (PbO_2_, 97%) was supplied by Shanghai Aladdin Biochemical Technology Co. Ltd. Nickel foam (NF) was acquired from Changsha Lyrun New Material Co., Ltd. High-purity water was obtained from Milli-Q purification system.

**Fabrication of** **CsPbX_3_@AlPO-5 composite**

AlPO-5 zeolite was synthesized by solvothermal method of the crystallization at 180 ℃ for 72 h. The CsPbX_3_ perovskite precursor solution was obtained by dissolving equimolar ratios of CsX and PbX_2_ in 0.1 M of DMSO solvent. Subsequently, AlPO-5 zeolite was immersed in precursor solution accompanied by sonication for 30 min. Afterward, the excess perovskite solution was removed through filtration under low pressure. Finally, CsPbX_3_@AlPO-5 composite was formed by thermal annealing at 100 ℃ for 30 min.

**Physicochemical characterizations**

X-ray diffraction (XRD) patterns were collected on a Rigaku Smartlab-9kW diffractometer (Cu *K*_α_ radiation, *λ* = 1.5406 Å) at a scan speed of 2 ^o^/min. Grazing incidence X-ray diffraction (GIXRD) data were recorded with an incidence angle of 0.3^o^. Transmission electron microscopy (TEM) images, high-resolution transmission electron microscopic (HRTEM) images, high-angle annular dark field scanning transmission electron microscopy (HAADF-STEM) images were obtained using a JEM-2800 (JEOL) electron microscope operated at 200 kV. Energy dispersive spectrometry (EDS) was performed by a X-Max 80T from Oxford. The iDPC-STEM images were obtained in a Cs-corrected STEM (FEI Titan Cubed Themis G2 300) operated at 300 kV. Considering the lower crystallinity of zeolite fragments when CsPbBr_3_@AlPO-5 composite undergoes water oxidation, CsPbBr_3_@S-1 was used in iDPC-STEM test. The chemical states of the prepared samples were analyzed by X-ray photoelectron spectrometer (XPS, Thermo Fisher Scientific, ESCALAB Xi+) with monochromatic Al *K*_α_ as radiation source. UV-vis absorption spectra were measured on a UV-vis spectrophotometer (HITACHI-UH4150). Fourier transform infrared (FTIR) spectrum was acquired on a FTIR spectrometer (Bruker, VERTEX 70). Inductively coupled plasma (ICP) analysis was carried out using an ICAP 6300 of Thermo scientific. The chemical state and configuration of lead were investigated by X-ray absorption spectroscopy (XAS) from BL11B beamline of Shanghai Synchrotron Radiation Facility (SSRF). Impedance analyzer (Solartron 1260) was employed to examine the proton conductivity in a frequency window from 1 (or 10) Hz to 1 MHz. Nitrogen adsorption-desorption isotherms were determined by the Micromeritics ASAP 2020 instrument based on the Brunauer-Emmett-Teller (BET) equation.

**Electrochemical measurements**

Electrochemical experiments were carried out using CHI Instruments (CHI 760E, China) in a standard three-electrode setup, of which 1 M KOH solution was used as the electrolyte, a platinum sheet and Hg/HgO electrode (in 1 M KOH, 0.098 V_SHE_) was employed as the counter-electrode and reference electrode, respectively. A piece of nickel foam (1.2 cm × 1 cm) covered with a thin catalyst film was employed as the working electrode. Briefly, a homogeneous paste was obtained comprised of 5 mg electroactive material (containing roughly 0.5 mg of CsPbX_3_ NCs) and several drops of Nafion solution (60 μl). Then the paste was embedded on the surface of the open-cell nickel foam (NF) yielding a coating area of 1 cm^2^ (1 cm × 1 cm) and dried at 60 ℃ for 5 h. Note that before loading the catalyst, the purchased NF was carefully cleaned with acetone, ultra-purified water and ethanol assisted by ultrasound for 30 min to remove the surface impurities. The electrolyte was degassed by high-purity anhydrous oxygen (O_2_, 99.99%) for at least 30 min prior to the electrochemical tests and maintained with O_2_ flow throughout the test period. Before and after electrochemical measurements in KOH solutions with varying pH, potential calibration of the Hg/HgO reference electrode was performed relative to a second Hg/HgO electrode that is solely stored. No potential drift was observed after such experiments, indicating the validity of the 0.098 V versus the standard hydrogen electrode (SHE) for Hg/HgO reference electrode in this study.

All potentials were calibrated versus to the reversible hydrogen electrode (RHE) based on the following equation:

*E*(RHE) = *E*(Hg/HgO) + 0.05916 × pH + 0.098 V (1)

Besides, compensation of *iR*_s_ drop of each polarization curve with ninety percent was automatically performed by the electrochemical workstation. Cyclic voltammetry (CV) measurements were conducted with different scanning rates. Linear sweep voltammetry (LSV) curves were collected at a constant rate of 5 mV·s^-1^, and Tafel slopes were given by fitting the linear part of overpotential versus current density in log scale (log |*j*|).

Electrochemical impedance spectroscopy (EIS) was measured within frequency window ranging from 100 KHz to 0.01 Hz with an amplitude of 5 mV. The electrochemical surface areas (ECSA) were investigated by CV method measuring the current related to double-layer capacitance (*C*_dl_) in a non-Faradic potential window. Typically, the potentials were swept between 1.125 V_RHE_ and 1.225 V_RHE_ under different scan rates (10, 20, 30, 40, 50, 60, 70, 80, 90 and 100 mV·s^-1^). Then, the *C*_dl_ was determined by plotting the ∆*j* (*j*_a_ - *j*_c_) at 1.175 V_RHE_ against the scan rate.

The contribution of pseudocapacitive effects versus diffusion behaviors during the chemical restructuring process of CsPbX_3_@AlPO-5 composite can be qualitatively analyzed based on the relationship between the current (*i*) and scan speed (*v*) from CV curves:

*i* = *av^b^* (2)

Where *a* and *b* are adjustable parameters, and the gradient of the linear fit of the log(*i*) versus log(*ν*) plot is *b*^1^. The property of the kinetic reaction can be determined by the limiting cases of the *b-*value. Specifically, the *b*-value of 0.5 indicates a completely diffusion-controlled behavior, whereas a *b*-value of 1.0 means an ideal surface-controlled process including a pseudocapacitive-type intercalation mechanism. In addition, the percentage between surface-controlled and diffusion-controlled behavior can be quantified by separating the current (*i*) under a fixed potential (V) into capacitive current (linear relationship with the scan rate *v*) and diffusion-controlled one (proportional to the square root of the sweep rate):

*i*(V) = *k*_1_*v* + *k*_2_*v*^1/2^ (3)

By plotting *i*(V)/*v*^1/2^ versus *v*^1/2^, *k*_1_ and *k*_2_ are obtained from the gradient and the Y-intercept of a straight line.

Open circuit potential (OCP) decay was recorded by first activating the working electrode at 1.48 V_RHE_ for 2 min, followed by collecting the decay curve at open-circuit condition. In particular, one hour of surface oxidation of CsPbX_3_@AlPO-5 composite was achieved by holding open circuit condition of 58 min enabling steady state after the polarizing process at 1.48 V_RHE_, while two-hour oxidation would be realized when such processes were repeated twice in succession.

The stability of the water oxidation was evaluated by chronopotentiometry measurement at 10 mA·cm^-2^. The gaseous products of overall water splitting were monitored in real time by an on-line gas chromatograph. The Faradic Efficiency (FE) was obtained according to equation 4:

FE = (*m* × n × F) / (*I* × *t*) (4)

Where *m* is produced moles of oxygen or hydrogen (mol), n is the number of transfer electrons, F is Faraday constant (96485.33 C/mol), *I* is the given current density (A/cm^2^), and *t* is reaction time.

**Computational models and methods**

All the calculations were carried out by means of spin-polarized density functional theory (DFT) methods using Vienna Ab-initio Simulation Package (VASP)^2-4^. The exchange and correlation energies were described by the generalized gradient approximation (GGA) with the Perdew-Burke-Ernzerhof (PBE) functional^5,6^. The projector augmented-wave (PAW) method was used to describe the electron-ion interactions^7,8^. Based on our careful convergence tests, the plane wave energy cutoff was set to 400 eV. The convergence criterion of electronic structure was set to 10^-6^ eV, and the atomic relaxation was continued until the forces acting on atoms were smaller than 0.02 eV/Å. The Brillouin zone was sampled with 2 × 2 × 1 Monkhorst-Pack *k*-point mesh, and a Gaussian smearing of 0.05 eV is applied to speed up electronic convergence. The 2 × 2 supercell of α-PbO_2_ (200) and CsPbBr_3_ (100) surfaces were used to build the pristine PbO_2_ and CsPbBr_3_/α-PbO_2_ calculation models. A vacuum height of 20 Å along the vertical direction was selected to avoid the unwanted interaction between the slab and its period images. The final structure was illustrated with VESTA software^9^.

**Supplementary Figures**

**
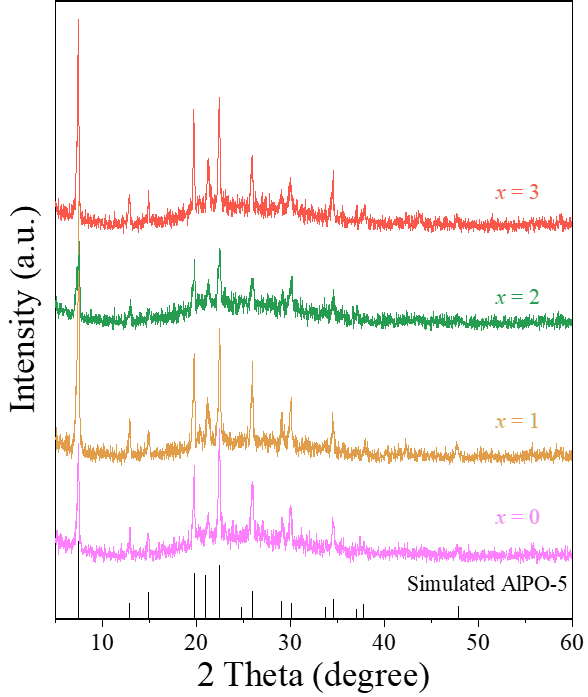
**

**Figure S1.** XRD patterns of prepared CsPbBr*_x_*I_3-_*_x_*@AlPO-5 composites (*x* = 0, 1, 2, 3).

**
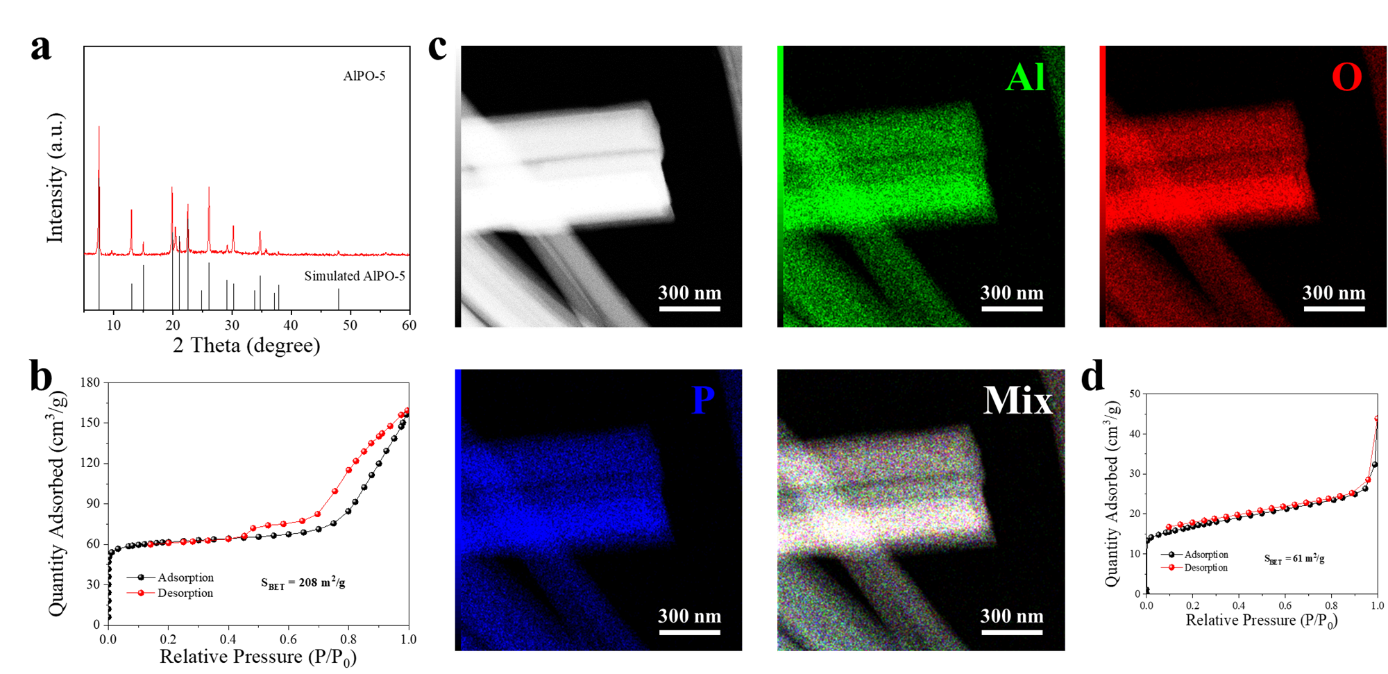
**

**Figure S2.** (a) XRD patterns, (b) nitrogen adsorption-desorption isotherms, and (c) HAADF-STEM image and elemental mappings of AlPO-5 zeolite. (d) nitrogen adsorption-desorption isotherms of CsPbBr_3_@AlPO-5 composite.

**Note:** XRD patterns display the characteristic diffraction peaks of pristine AFI-type zeolite, which is in good agreement with the simulated results of AlPO-5 zeolite, demonstrating the neat AlPO-5 matrix of the fabricated zeolite. Using the BET method, the specific surface area of AlPO-5 zeolite is determined to be 208 m^2^/g. In addition, the HAADF-STEM and element mapping images exhibit that the elements Al, O, and P are homogeneously distributed in the AlPO-5 zeolite. For comparison, the specific surface area of the CsPbBr_3_@AlPO-5 is much lower than that of neat AlPO-5, manifesting the pore channel in the zeolite are mainly blocked by guest CsPbBr_3_ NCs.


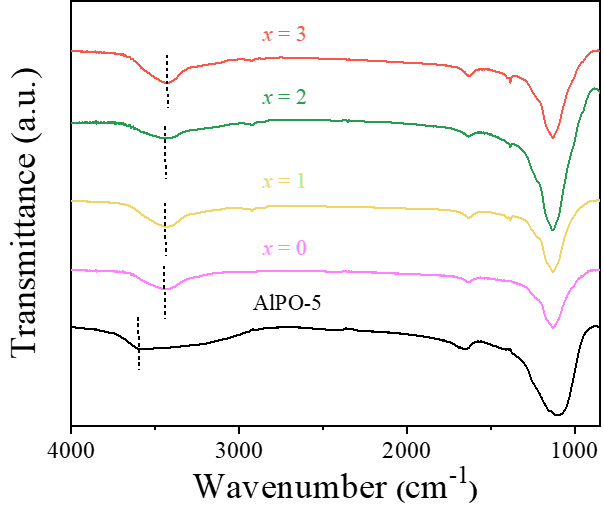


**Figure S3.** Fourier Transform Infrared (FT-IR) spectra of CsPbBr*_x_*I_3-_*_x_*@AlPO-5 composites (*x* = 0, 1, 2, 3) and AlPO-5 zeolite.


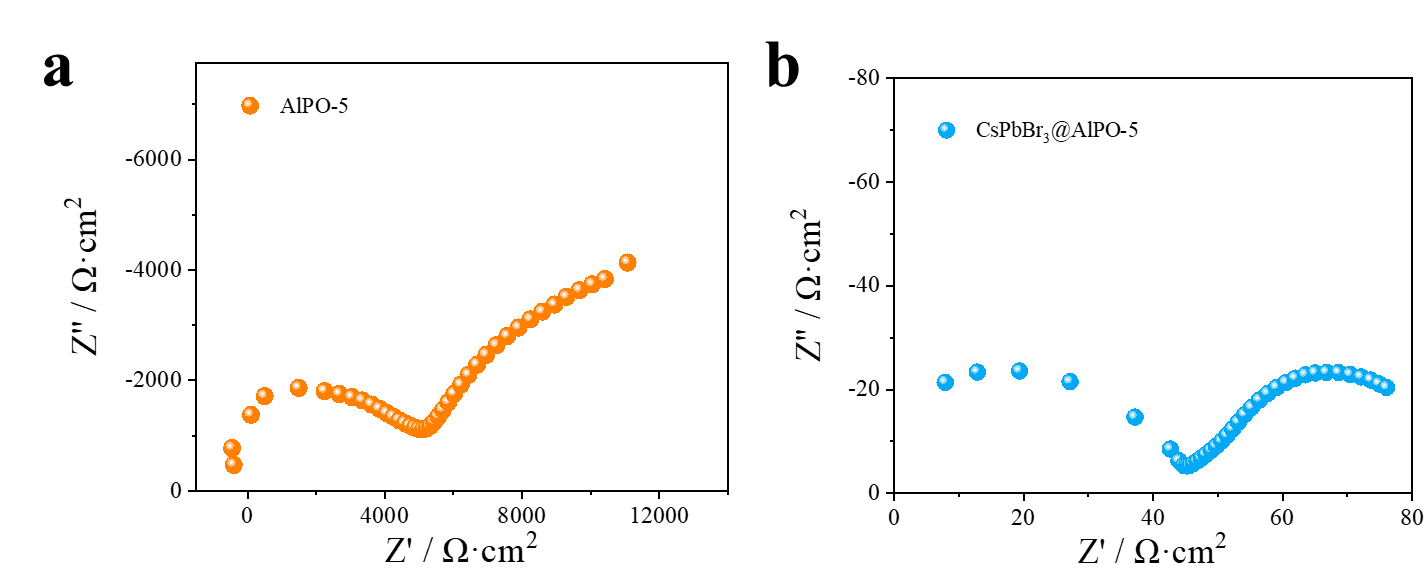


**Figure S4.** Nyquist plots of (a) AlPO-5 zeolite and (b) CsPbBr_3_@AlPO-5 composite.


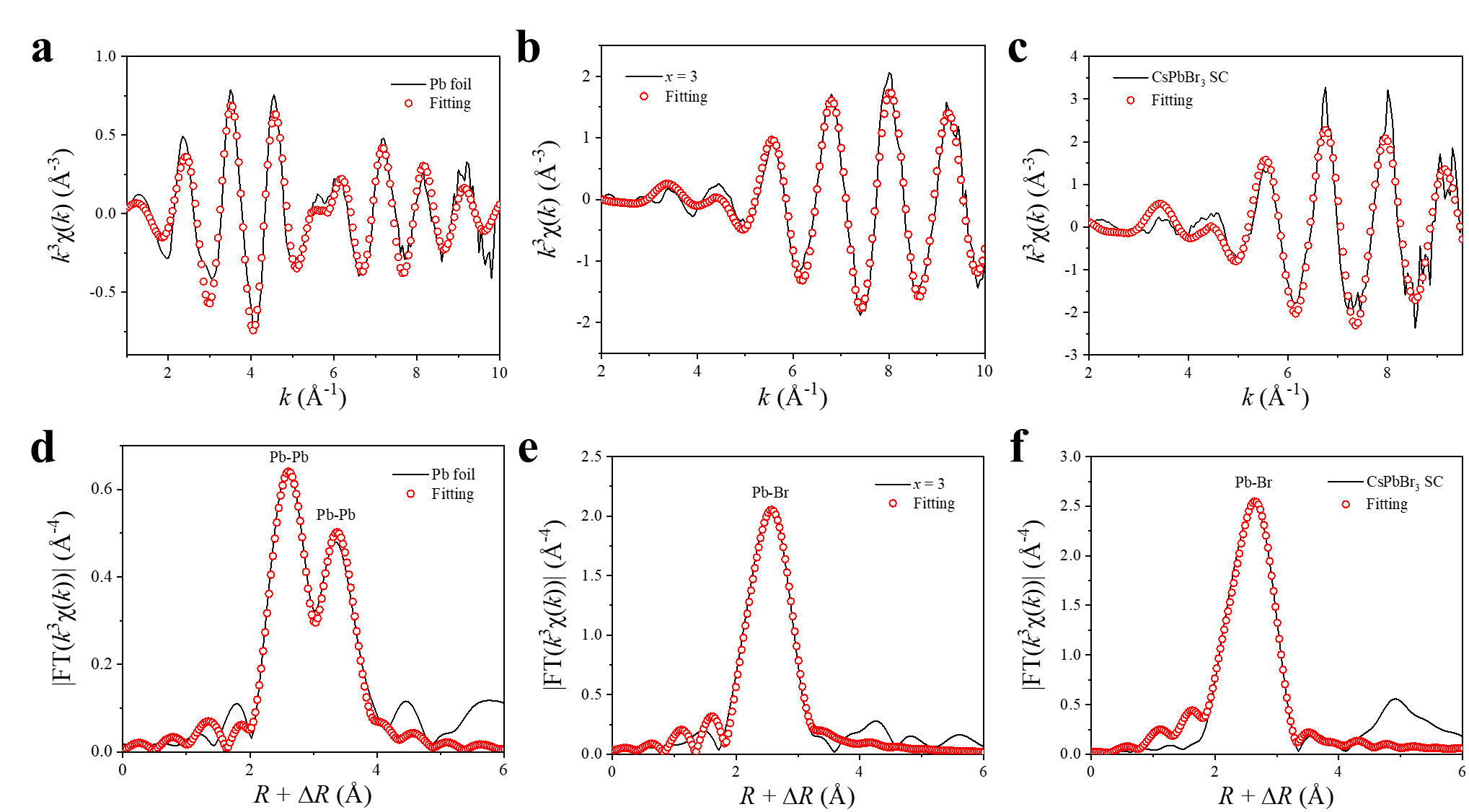


**Figure S5.** Pb L_3_-edge EXAFS oscillation functions *k*^3^χ(*k*) of (a) Pb foil, (b) CsPbBr_3_@zeolite, and (c) CsPbBr_3_ single crystal (SC). Fourier-transformed *k*^3^-weighted EXAFS spectra of (d) Pb foil, (e) CsPbBr_3_@zeolite, and (f) CsPbBr_3_ SC.


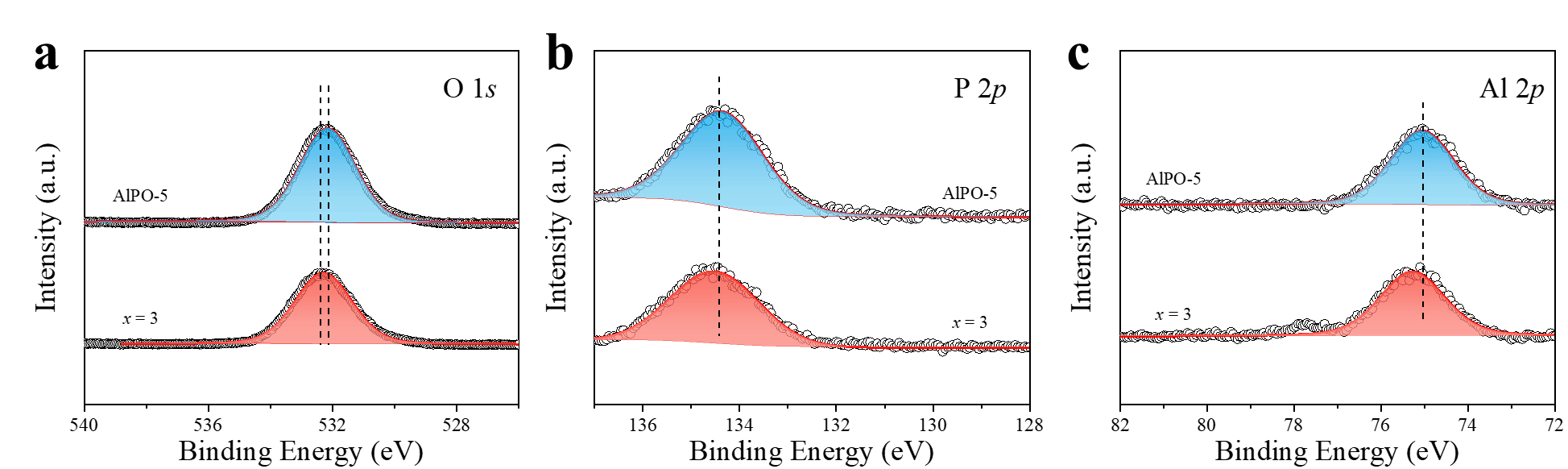


**Figure S6.** XPS spectra of (a) O 1*s*, (b) P 2*p*, and (c) Al 2*p* for AlPO-5 zeolite and CsPbBr_3_@AlPO-5 composite.


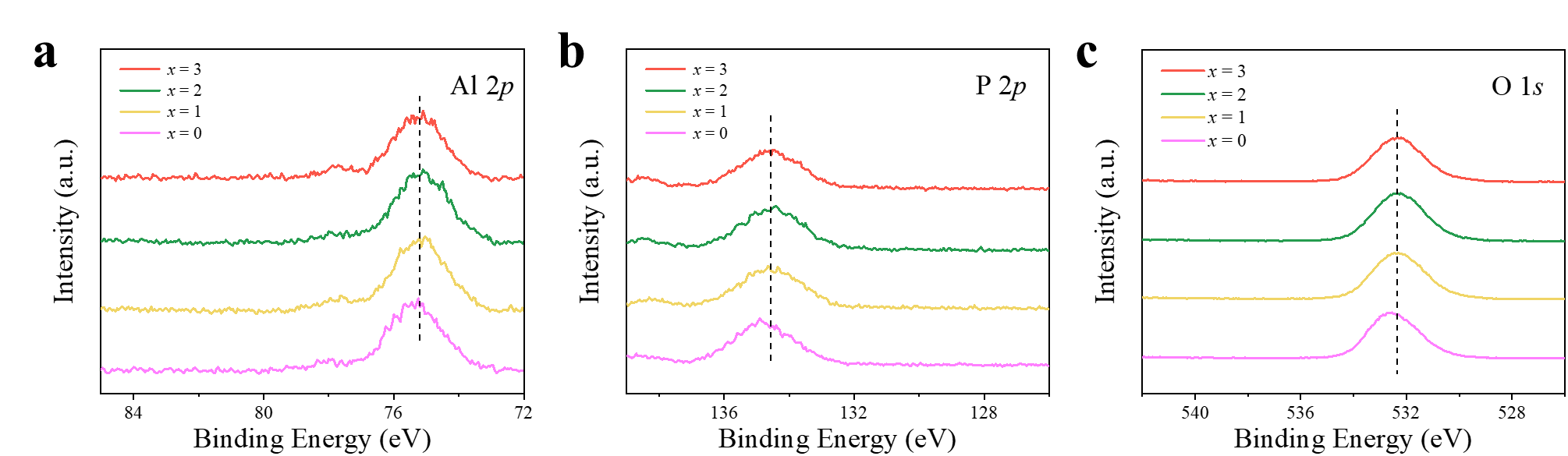


**Figure S7.** XPS spectra of (a) Al 2*p*, (b) P 2*p*, and (c) O 1*s* for CsPbBr*_x_*I_3-_*_x_*@AlPO-5 composites (*x* = 0, 1, 2, 3).

**Figure S8.** Polarization curves of CsPbBr_3_@AlPO-5, CsPbI_3_@AlPO-5, MAPbBr_3_@AlPO-5 composites and IrO_2_@NF.

**Note:** The CsPbBr_3_@AlPO-5 delivers an overpotential of 357 mV to achieve a current density of 100 mA·cm^-2^, smaller than that of the CsPbI_3_@AlPO-5 (480 mV). In our previous reports^10^, due to the triggered single-metal-site mechanism (SMSM), MAPbBr_3_@AlPO-5 composite require an overpotential of 321 mV to reach 100 mA·cm^-2^. Given the preferential oxygen-vacancy-site mechanism (OVSM) pathway of CsPbBr_3_@AlPO-5 (Fig. 5f-i, as discussed later), we find that the A-site elements of halide perovskites determine the LOM mechanism of the surface α-PbO_2_ active layer, which both outperforms IrO_2_@NF electrode.


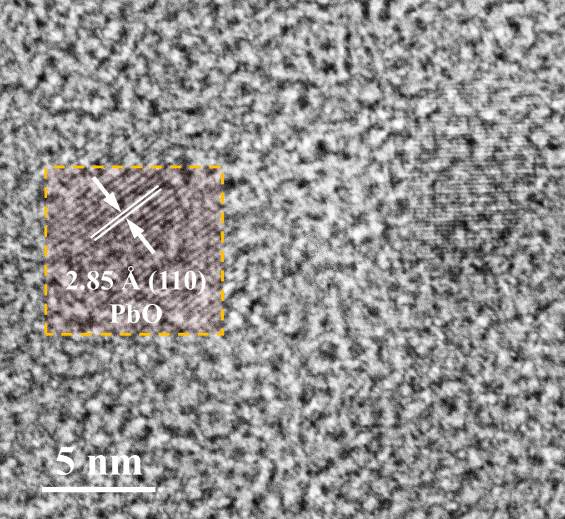


**Figure S9.** HRTEM image of CsPbBr_3_@AlPO-5 soaked in 1 M KOH for three hours.

**Note:** After the CsPbBr_3_@AlPO-5 was soaked in 1 M KOH for three hours, the HRTEM image manifests lattice spacing of 2.85 Å corresponding to the (110) facet of PbO, which further indicates that the valence state of Pb remains unchanged without applying an oxidation potential.


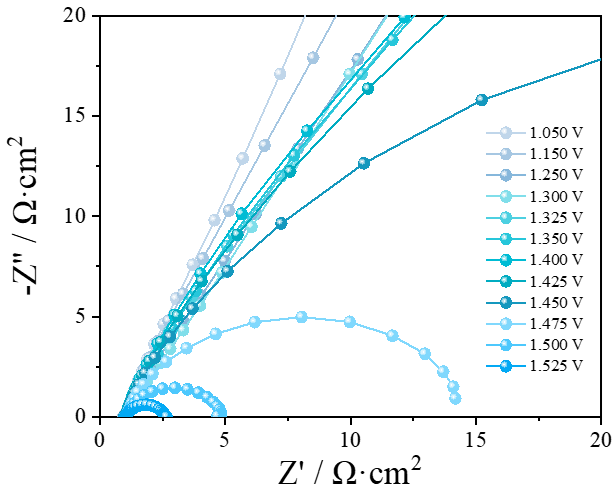


**Figure S10.** Nyquist plots of CsPbBr_3_@AlPO-5 composite at different applied potentials versus RHE in 1 M KOH.


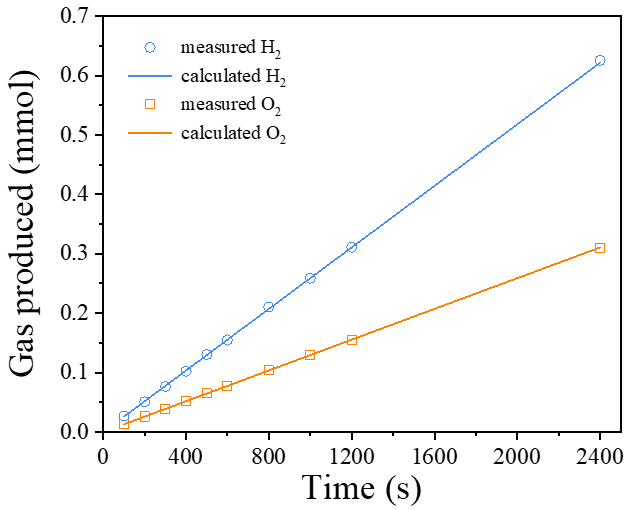


**Figure S11.** Comparison of experimental and theoretical yields of H_2_ and O_2_ produced for CsPbBr_3_@AlPO-5. The measurement was performed at 1.57 V_RHE_ in Ar-saturated 1 M KOH solution.


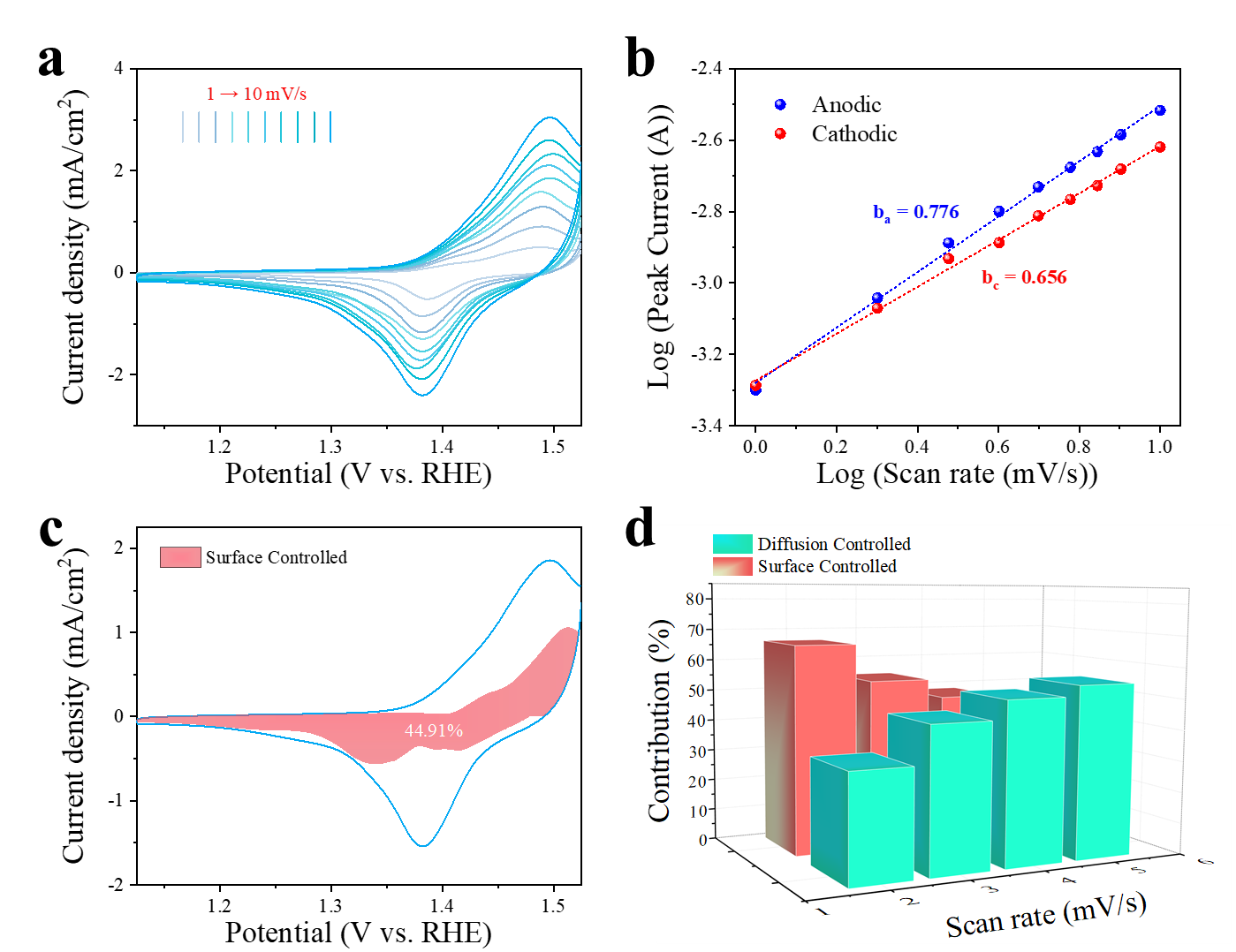


**Figure S12.** Kinetic analysis of CsPbI_3_@AlPO-5 composite. (a) CV curves from 1 to 10 mV/s; (b) The relationship between peak currents and scan rates from 1–10 mV/s; (c) Capacitive contribution at 5 mV/s; (d) The percentages of capacitive and diffusion-controlled contribution at different scan rates.

**Note:** For CsPbI_3_@AlPO-5, the *b* value of anodic peak current is 0.776 while that of cathodic peak current is 0.656 (Figure S12b), suggesting a poor reversibility behavior of the redox reaction of Pb^2+^/Pb^4+^. Further, the surface-controlled behaviour of CsPbI_3_@AlPO-5 contributes 66.38%, 54.41%, 48.35%, and 44.91% of the total charge at 2, 3, 4 and 5 mV/s, respectively, which demonstrates that the kinetics are increasingly dominated by diffusion-controlled behavior at lower scan rates. This also suggests that the inferior OER activity of CsPbI_3_@AlPO-5 is originated from the sluggish diffusion of OH^-^ ions.


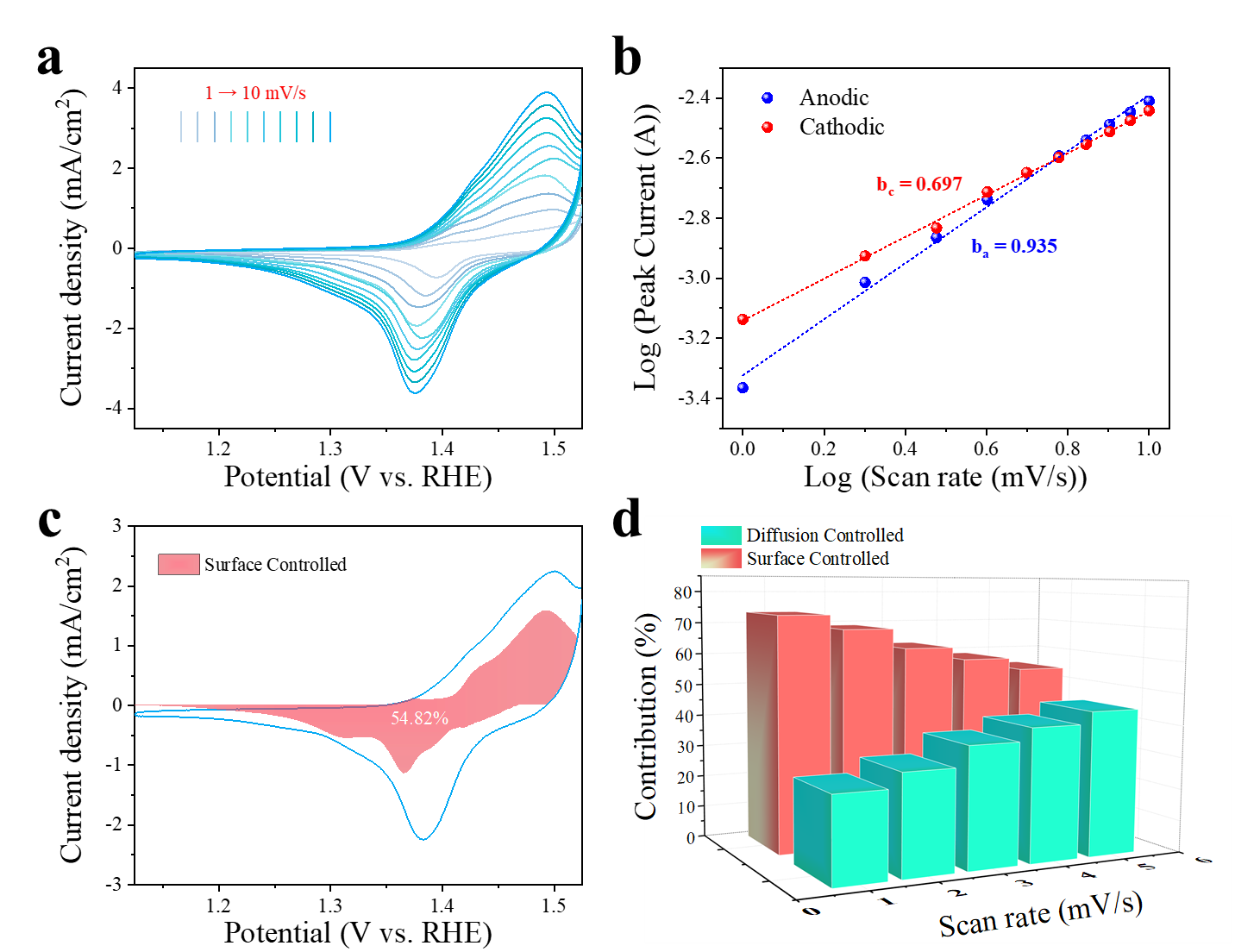


**Figure S13.** Kinetic properties of CsPbI_2_Br@AlPO-5 composite. (a) CV curves from 1 to 10 mV/s; (b) The relationship between peak currents and scan rates; (c) Capacitive contribution at a scan rate of 5 mV/s; (d) The percentages of capacitive and diffusion-controlled contribution at various scan rates.

**Note:** The *b* value obtained from the anodic and cathodic peaks are 0.935 and 0.697, respectively, reflecting the dominant surface-controlled behaviour that features the lowest reversibility in the redox reaction from 1–10 mV/s. Additionally, at a sweep rate of 5 mV/s, 54.82% of the redox current arises from the surface-controlled behaviour for CsPbI_2_Br@AlPO-5 composite, as shown in Figure S13d.


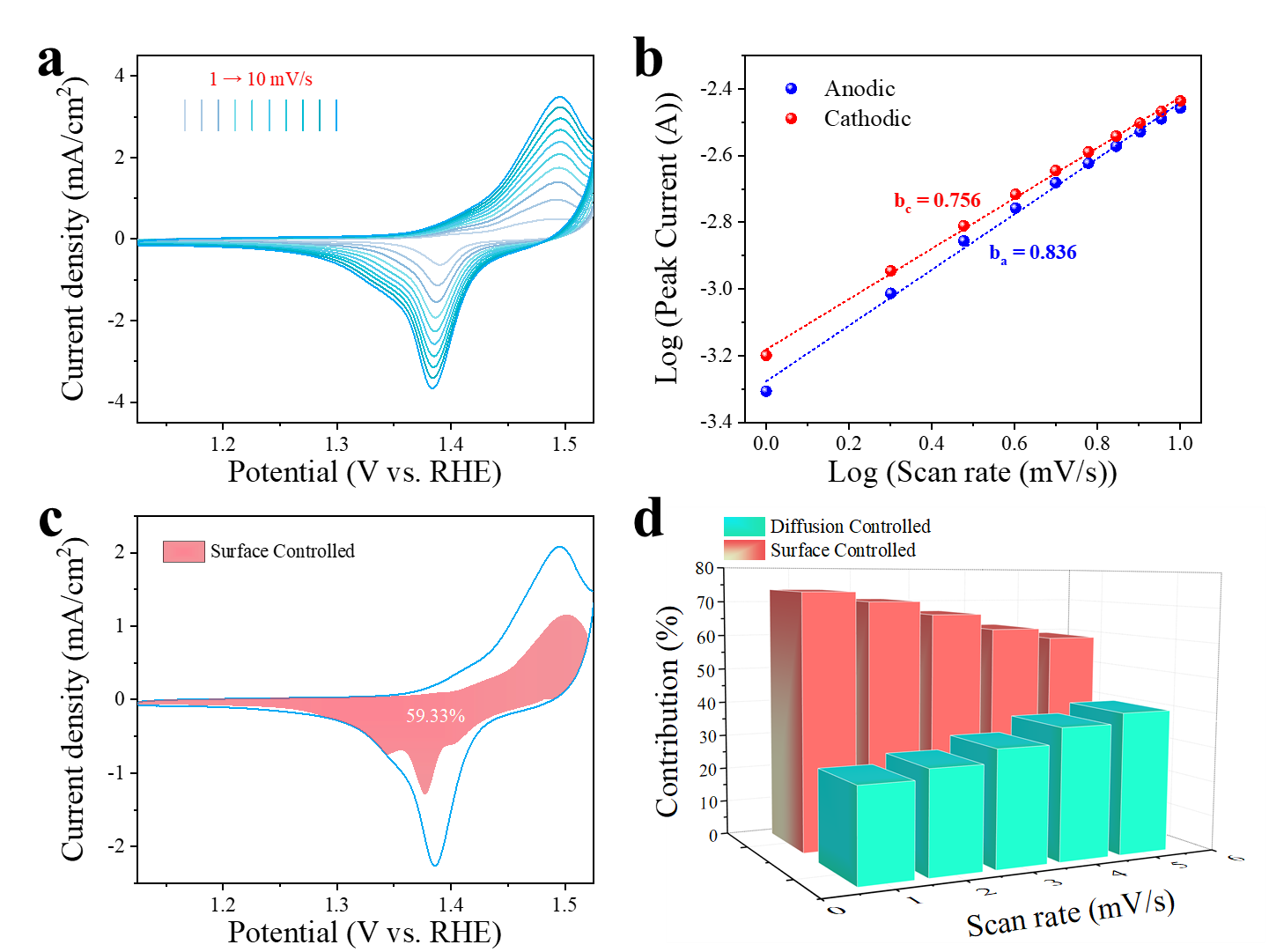


**Figure S14.** Kinetic properties of CsPbIBr_2_@AlPO-5 composite. (a) CV curves from 1 to 10 mV/s; (b) The relationship between peak currents and scan rates; (c) Capacitive contribution at 5 mV/s; (d) The percentages of capacitive and diffusion-controlled contribution at various scan rates.

**Note:** For the CsPbIBr_2_@AlPO-5, the redox reaction of Pb^2+^/Pb^4+^ exhibits better electrochemical reversibility compared to CsPbI_2_Br@AlPO-5 and CsPbI_3_@AlPO-5 composites, with *b*-values for the anodic and cathodic peaks of 0.836 and 0.756 (1–10 mV/s), respectively, which indicates that the reconstruction kinetics are mainly surface-controlled. Also, the pseudocapacitive behavior contributes 73.72%, 70.86%, 66.95%, 62.33%, and 59.33% of the total current at 1, 2, 3, 4 and 5 mV/s, respectively.


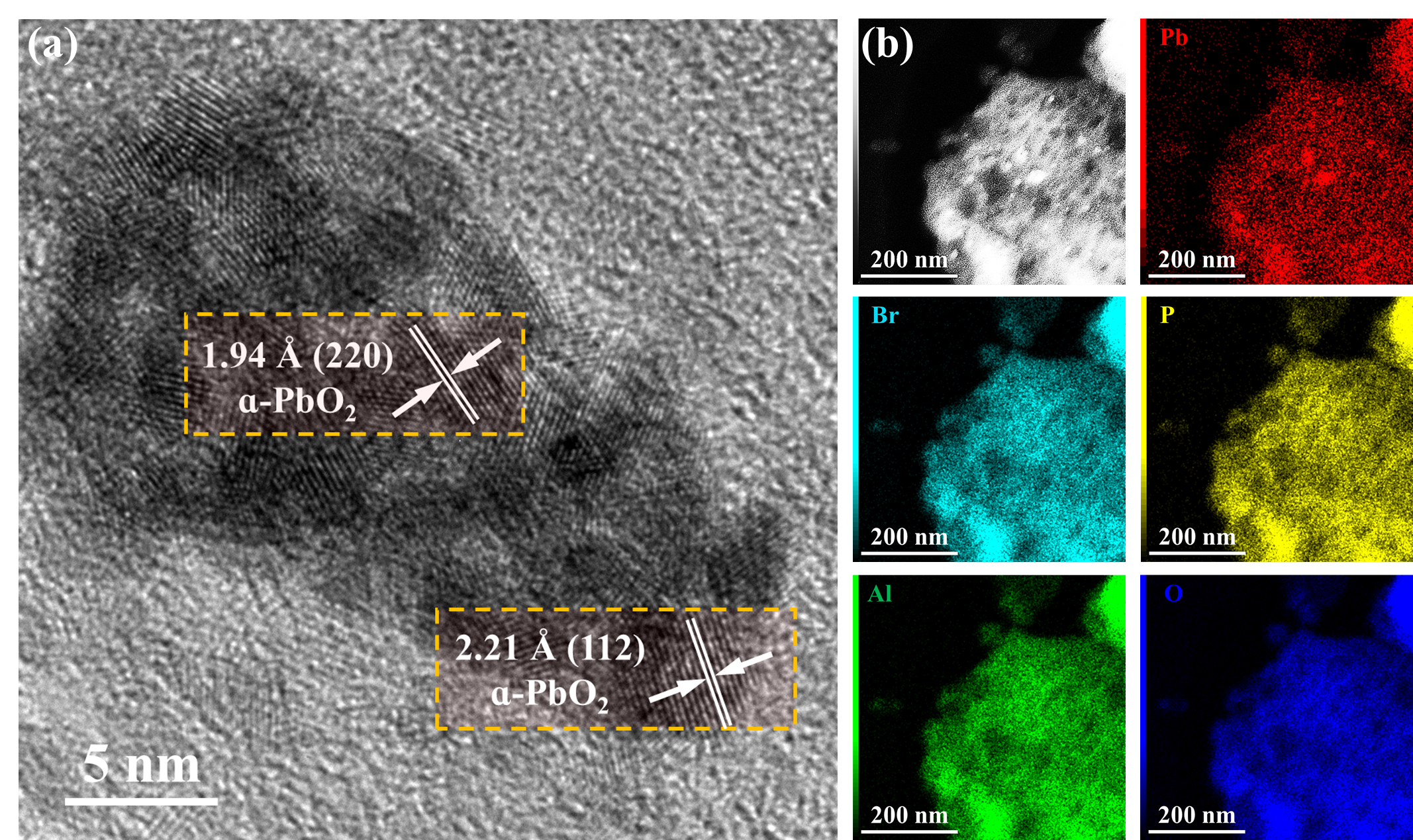


**Figure S15.** (a) HRTEM image and (b) elemental mappings of CsPbBr_3_@AlPO-5 after the 40-hour water oxidation.

**Note:** After 40 h of water oxidation, HRTEM observation displays lattice fringes corresponding to the (220) and (112) facets of *α*-PbO_2_. Further, the elemental mappings demonstrate the homogeneous distributions of Al, P, O, Br, and Pb in the CsPbBr_3_@AlPO-5, which suggests that the reconstructed CsPbBr_3_/α-PbO_2_ and zeolite fragments of CsPbBr_3_@AlPO-5 composite are well retained after stability test.

**Figure S16.** GIXRD patterns of CsPbBr_3_@AlPO-5 composite after the OER.

**Note:** After water oxidation, the diffraction peak at 30.20° is ascribed to the (200) crystal planes of perovskite CsPbBr_3_, while the diffraction peaks centered at 24.20°, 31.42°, and 39.40° arise from the retained zeolite matrices. In addition, the diffraction peak located at 34.10° is assigned to the *α*-PbO_2_ phase, which further verifies the reconstructed CsPbBr_3_/α-PbO_2_ and zeolite fragments of CsPbBr_3_@AlPO-5 composite after OER test.


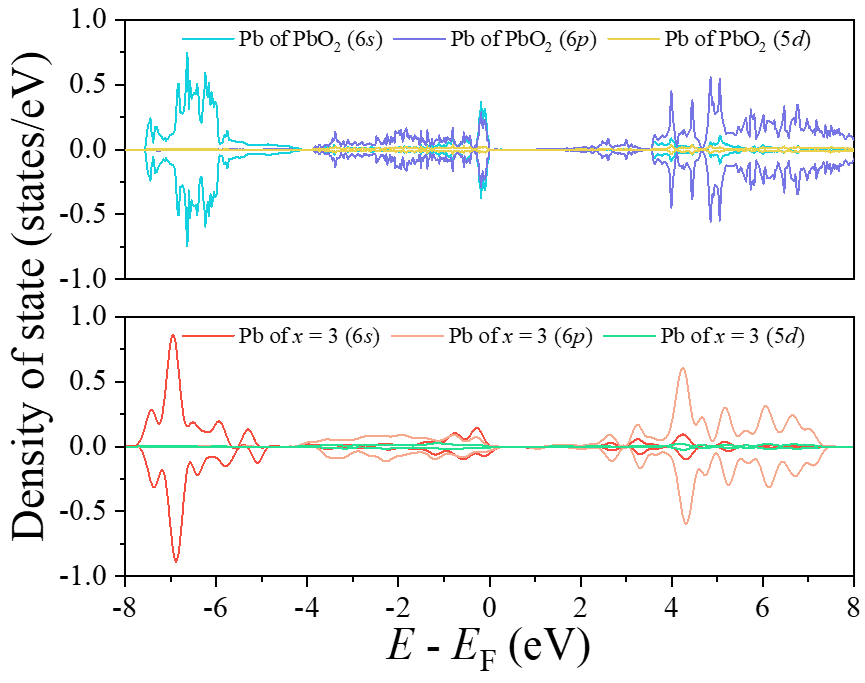


**Figure S17.** Projected density of states (DOS) of Pb 5*d*, 6*s*, and 6*p* orbitals of surface α-PbO_2_ in CsPbBr_3_/α-PbO_2_ and pure α-PbO_2_.


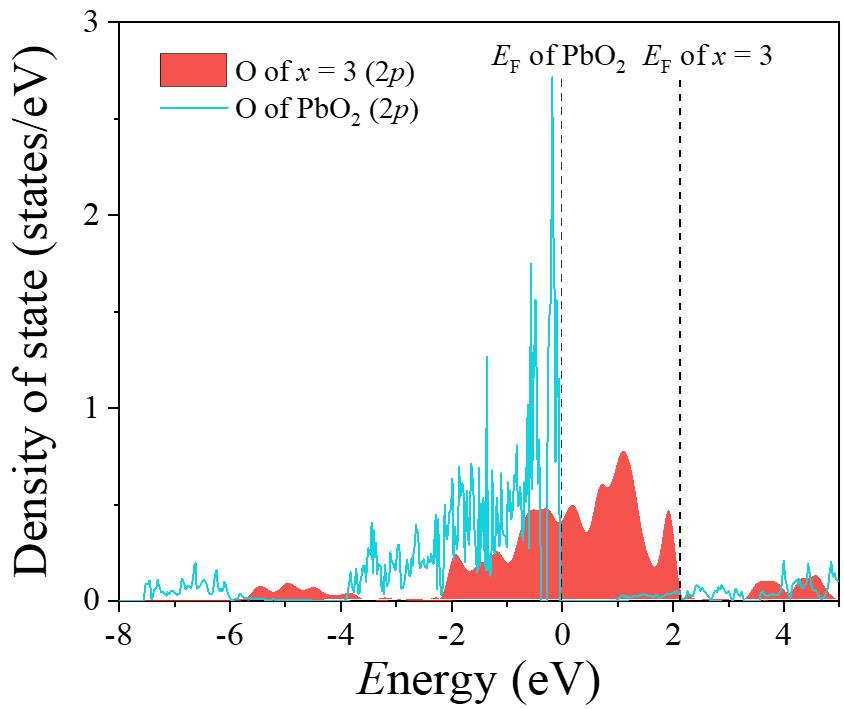


**Figure S18.** Projected DOS of O 2*p* orbitals of surface α-PbO_2_ in CsPbBr_3_/α-PbO_2_ and pure α-PbO_2_. The abscissa is relative to the vacuum level, and the Fermi level (*E*_F_) of pure α-PbO_2_ is set to zero-point.


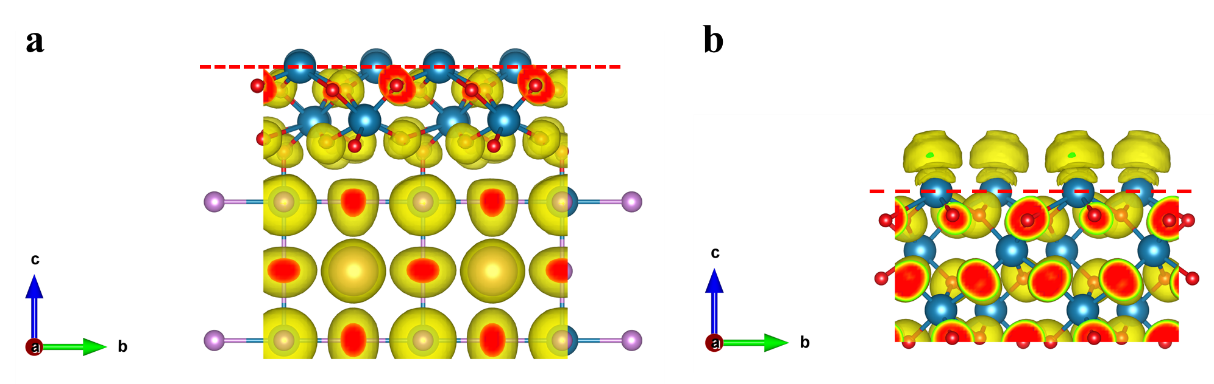


**Figure S19.** Electron localization function (ELF) of (a) CsPbBr_3_/α-PbO_2_ and (b) α-PbO_2_. (Dark blue, red, purple, and orange balls indicate lead, oxygen, bromine, and cesium atoms, respectively.)

**Note:** The distance of Pb-O bond along the *c*-axis in surface α-PbO_2_ of CsPbBr_3_/α-PbO_2_ is approximately 0.77 Å, lower than that of in pristine α-PbO_2_ (~1 Å).


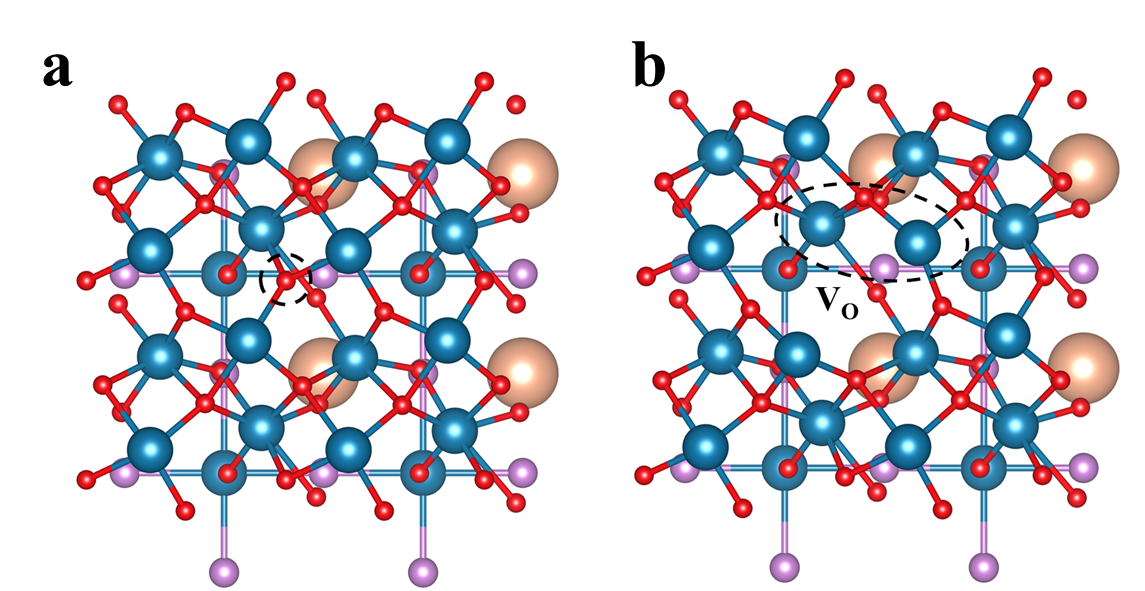


**Figure S20.** Local configurations of (a) pristine CsPbBr_3_/α-PbO_2_ and (b) CsPbBr_3_/α-PbO_2_ with a single oxygen vacancy (V_O_) located in surface bridging-oxygen. (Dark blue, red, purple, and orange balls represent lead, oxygen, bromine, and cesium atoms, respectively.)

**Note:** The corresponding V_O_ formation energy is -0.17 eV, much smaller than that for pure α-PbO_2_ with a value of 0.91 eV.


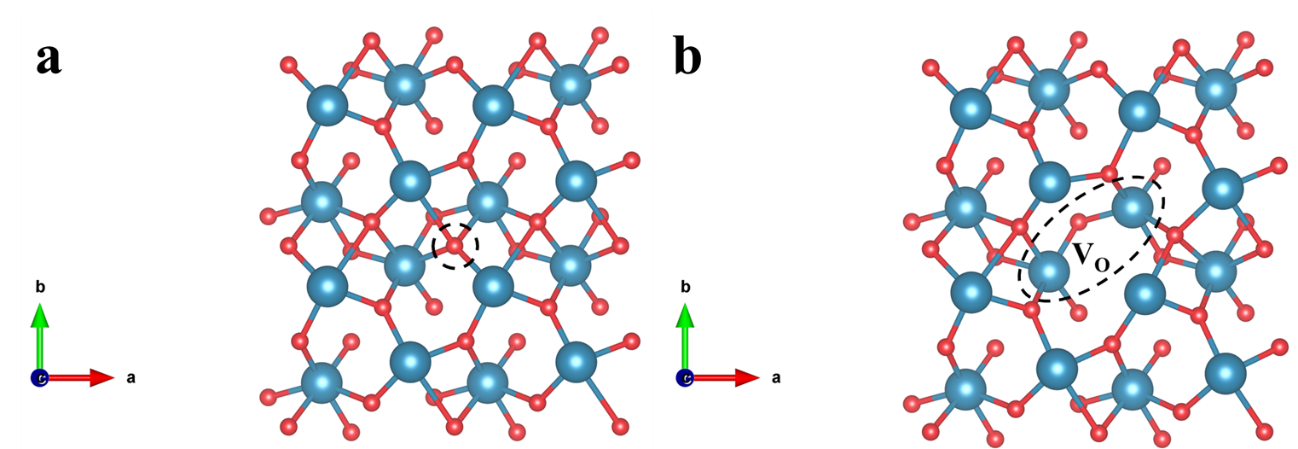


**Figure S21.** Local configurations of (a) pristine α-PbO_2_ and (b) α-PbO_2_ with a single V_O_ located at surface bridging-oxygen. (Dark blue and red balls stand for lead and oxygen atoms, respectively.)

**Figure S22.** Polarization curves of composites with different precursors.

**Note:** When changing the ratio of the precursor solution, the composites display poor OER performance compared with CsPbBr_3_@AlPO-5 composite, which further confirms the key role of CsPbBr_3_ perovskites in steering the electronic states of surface α-PbO_2_, achieving higher intrinsic OER activity.

**Supplementary Tables**

**Table S1.** ICP-MS results of CsPbBr_3_@AlPO-5 composite.

| Sample state | Element | Atomic percentage% |
| --- | --- | --- |
| pristine | Br | 74.12 |
|  | Pb | 25.88 |
| after water oxidation | Br | 48.65 |
|  | Pb | 51.35 |

**Table S2.** Proton conductivity of AlPO-5 zeolite and CsPbBr_3_@AlPO-5 composite.

| Sample | Proton conductivity (S·cm^-1^) |
| --- | --- |
| AlPO-5 | 5.88 × 10^-6^ |
| CsPbBr_3_@AlPO-5 | 8.89 × 10^-4^ |

**Table S3.** Fitted parameters of Pb L_3_-edge EXAFS curves of CsPbBr_3_ SC and CsPbBr_3_@zeolite composite. (*^a^CN*: coordination numbers; *^b^R*: bond distance; *^c^σ*^2^: Debye-Waller factors; *^d^R* factor indicates the goodness of the fit.)

| Sample | bond | *^a^CN* | *^b^R*(Å) | *^c^σ*^2^(Å^2^) | *^d^R* factor |
| --- | --- | --- | --- | --- | --- |
| Pb foil | Pb-Pb | 12.0 | 3.398±0.009 | 0.0245 | 0.0076 |
| CsPbBr_3_ SC | Pb-Br | 6.0±0.5 | 2.980±0.005 | 0.0175 | 0.0023 |
| CsPbBr_3_@zeolite | Pb-Br | 5.1±0.5 | 2.938±0.005 | 0.0119 | 0.0036 |

**Table S4.** Centroid positions of oxygen, aluminum, and phosphorus core level peaks for AlPO-5 zeolite and CsPbBr_3_@AlPO-5 composite.

| Sample | O 1*s* | Al 2*p* | P 2*p* |
| --- | --- | --- | --- |
| AlPO-5 | 532.16 eV | 75.05 eV | 134.35 eV |
| CsPbBr_3_@AlPO-5 | 532.40 eV | 75.28 eV | 134.51 eV |

**Table S5.** Fitted parameters of the equivalent circuits of CsPbBr*_x_*I_3-_*_x_*@AlPO-5 composites (*x* = 0, 1, 2, 3).

| sample | *R*_s_/Ω·cm^2^ | C/mF·cm^-2^ | *R*_p_/Ω·cm^2^ | CPE/mF·cm^-2^ | *R*_ct_/Ω·cm^2^ |
| --- | --- | --- | --- | --- | --- |
| *x* = 0 | 1.405 | 6.11 | 77.66 | 7.67 | 2.095 |
| *x* = 1 | 1.231 | 6.99 | 41.88 | 7.73 | 1.507 |
| *x* = 2 | 1.415 | 3.10 | 25.75 | 5.07 | 1.540 |
| *x* = 3 | 1.185 | 12.97 | 14.96 | 10.96 | 0.105 |

**Table S6.** Fitted parameters of equivalent circuit of CsPbBr_3_@AlPO-5 at different potentials.

| Equivalent circuit | *E*_RHE_ /V | *R*_s_/Ω·cm^2^ | CPE_1_/mF·cm^-2^ | *R*_p_/Ω·cm^2^ | CPE_2_/mF·cm^-2^ | *R*_ct_/Ω·cm^2^ |
| --- | --- | --- | --- | --- | --- | --- |
| *R*_s_(*Q*_1_*R*_p_) | 1.050 | 1.353 | 1.33 | 17530 | -- | -- |
| *R*_s_(*Q*_1_*R*_p_) | 1.150 | 1.364 | 1.65 | 4153 | -- | -- |
| *R*_s_(*Q*_1_*R*_p_) | 1.250 | 1.404 | 2.84 | 1475 | -- | -- |
| *R*_s_(*Q*_1_*R*_p_) | 1.300 | 1.422 | 4.33 | 726 | -- | -- |
| *R*_s_(*Q*_1_*R*_p_) | 1.325 | 1.237 | 21.45 | 403 | -- | -- |
| *R*_s_(*Q*_1_*R*_p_) | 1.350 | 1.226 | 23.19 | 254 | -- | -- |
| *R*_s_(*Q*_1_*R*_p_) | 1.400 | 1.257 | 27.33 | 155 | -- | -- |
| *R*_s_(*Q*_1_*R*_p_)(*Q*_2_*R*_ct_) | 1.425 | 1.057 | 23.56 | 110.70 | 7.19 | 0.12 |
| *R*_s_(*Q*_1_*R*_p_)(*Q*_2_*R*_ct_) | 1.450 | 1.238 | 16.55 | 55.80 | 6.87 | 0.11 |
| *R*_s_(*Q*_1_*R*_p_)(*Q*_2_*R*_ct_) | 1.475 | 0.990 | 12.73 | 13.69 | 6.04 | 0.05 |
| *R*_s_(*Q*_1_*R*_p_)(*Q*_2_*R*_ct_) | 1.500 | 0.993 | 11.18 | 3.81 | 4.89 | 0.03 |
| *R*_s_(*Q*_1_*R*_p_)(*Q*_2_*R*_ct_) | 1.525 | 1.008 | 8.42 | 1.63 | 2.18 | 0.03 |

**Table S7.** Diffusion coefficient of hydroxide ions of CsPbBr_3_@AlPO-5 and MAPbBr_3_@AlPO-5.

| sample | Diffusion coefficient (cm^2^·s^-1^) |
| --- | --- |
| CsPbBr_3_@AlPO-5 | 5.0678 × 10^-7^ |
| MAPbBr_3_@AlPO-5 | 5.2112 × 10^-8^ |

**Table S8.** Comparison of OER performance of CsPbBr_3_@AlPO-5 composite with recently reported transition-metal electrocatalysts.

| Material | Electrolyte (KOH) | *η*_100_ (mV) | Substrate | Ref. |
| --- | --- | --- | --- | --- |
| Co_3_O_4_/CoFe_2_O_4_@NF | 1.0 M | 405 | NF | [S11] |
| Ni_3_S_2_/VS_4_ nano horn | 1.0 M | 375 | NF | [S12] |
| Ni-Mo_2_C/NC@NF | 1.0 M | 435 | NF | [S13] |
| Ru/NiFe LDH | 1.0 M | 380 | NF | [S14] |
| Fe_ads_-NiFeOOH | 1.0 M | 405 | CC | [S15] |
| La(Co_0.2_Mn_0.2_Fe_0.2_Ni_0.2_Cu_0.2_)O_3_ | 1.0 M | 445 | NF | [S16] |
| La(CrMnFeCo_2_Ni)O_3_ | 1.0 M | 400 | NF | [S17] |
| Ni/Mo_2_C(1:2)-NCNFs | 1.0 M | 440 | NF | [S18] |
| LaCoO_3_ | 1.0 M | 465 | NF | [S19] |
| CoMoNiS-NF-31 | 1.0 M | 385 | NF | [S20] |
| Co_0.8_Fe_0.2_ hydroxide | 1.0 M | 410 | NF | [S21] |
| La_2_Ni_0.5_Co_0.5_MnO_6_@MoO_3_/P | 1.0 M | 382 | NF | [S22] |
| Ni(CN)_2_/NiSe_2_ | 1.0 M | 480 | GC | [S23] |
| NiCoFe LDH | 1.0 M | 475 | CP | [S24] |
| LaCoO_3_/NF | 1.0 M | 408 | NF | [S25] |
| H-LFCMO@NF | 1.0 M | 380 | NF | [S26] |
| Co-P/HNCW-800 | 1.0 M | 470 | GC | [S27] |
| Fe_3_O_4_-70/NiB-CP | 1.0 M | 480 | CP | [S28] |
| CsPbBr_3_@AlPO-5 | 1.0 M | 357 | NF | This work |

**Note:** NF: Nickel foam; CC: Carbon cloth; GC: Glassy carbon; CP: Carbon paper.

**References for Supporting Information**

1 Chen, R. et al. Layered Structure Causes Bulk NiFe Layered Double Hydroxide Unstable in Alkaline Oxygen Evolution Reaction. *Adv. Mater.* **31**, 1903909, (2019).

2 Kresse, G. & Hafner, J. *Ab* initio molecular dynamics for liquid metals. *Phys. Rev.* **47**, 558-561, (1993).

3 Kresse, G. & Furthmüller, J. Efficient iterative schemes for *ab* initio total-energy calculations using a plane-wave basis set. *Phys. Rev.* **54**, 11169, (1996).

4 Kresse, G. & Furthmüller, J. Efficiency of ab-initio total energy calculations for metals and semiconductors using a plane-wave basis set. *Comput. Mater. Sci.* **6**, 15-50, (1996).

5 Perdew, J. P. & Wang, Y. Accurate and simple analytic representation of the electron-gas correlation energy. *Phys. Rev. B* **45**, 13244-13249, (1992).

6 Perdew, J. P., Burke, K. & Ernzerhof, M. Generalized gradient approximation made simple. *Phys. Rev. Lett.* **77**, 3865, (1996).

7 Blochl, P. E. Projector augmented-wave method. *Phys. Rev. B* **50**, 17953-17979, (1994).

8 Kresse, G. & Joubert, D. From ultrasoft pseudopotentials to the projector augmented-wave method. *Phys. Rev. B* **59**, 1758, (1999).

9 Momma, K. & Izumi, F. *VESTA* *3* for three-dimensional visualization of crystal, volumetric and morphology data. *J. Appl. Crystallogr.* **44**, 1272-1276, (2011).

10 Ren, X. R. et al. Surface Restructuring of Zeolite-Encapsulated Halide Perovskite to Activate Lattice Oxygen Oxidation for Water Electrolysis. *Adv. Mater.* **35**, 2301166, (2023).

11 Ma, Y. B. et al. Photothermal-Magnetic Synergistic Effects in an Electrocatalyst for Efficient Water Splitting under Optical-Magnetic Fields. *Adv. Mater.* 2303741, (2023).

12 Yang, D. et al. Formation of hierarchical Ni_3_S_2_ nanohorn arrays driven by in-situ generation of VS_4_ nanocrystals for boosting alkaline water splitting. *Appl. Catal. B: Environ.* **257**, 117911, (2019).

13 Xu, Z. X., Jin, S., Seo, M. H. & Wang, X. L. Hierarchical Ni-Mo_2_C/N-doped carbon Mott-Schottky array for water electrolysis. *Appl. Catal. B: Environ.* **292**, 120168, (2021).

14 Chen, Y. et al. Metastabilizing the Ruthenium Clusters by Interfacial Oxygen Vacancies for Boosted Water SplittingElectrocatalysis. *Adv. Energy Mater.* **14**, 2400059, (2024).

15 Zhang, Q. et al. Unraveling the Mechanism of Self-Repair of NiFe-Based Electrocatalysts by Dynamic Exchange of Iron during the Oxygen Evolution Reaction. *ACS Catal.* **13**, 14975-14986, (2023).

16 Meng, Z. S. et al. A general strategy for preparing hollow spherical multilayer structures of Oxygen-Rich vacancy transition metal Oxides, especially high entropy perovskite oxides. *Chem. Eng. J.* **457**, 141242, (2023).

17 Nguyen, T. X. et al. Advanced High Entropy Perovskite Oxide Electrocatalyst for Oxygen Evolution Reaction. *Adv. Funct. Mater.* **31**, 2101632, (2021).

18 Li, M. X. et al. Ni strongly coupled with Mo_2_C encapsulated in nitrogen‐doped carbon nanofibers as robust bifunctional catalyst for overall water splitting. *Adv. Energy Mater.* **9**, 1803185, (2019).

19 Hong, S. K. et al. Deep Eutectic Solvent Synthesis of Perovskite Electrocatalysts for Water Oxidation. *ACS Appl. Mater. Interfaces* **14**, 23277-23284, (2022).

20 Yang, Y. et al. Hierarchical Nanoassembly of MoS_2_/Co_9_S_8_/Ni_3_S_2_/Ni as a Highly Efficient Electrocatalyst for Overall Water Splitting in a Wide pH Range. *J. Am. Chem. Soc.* **141**, 10417-10430, (2019).

21 Wang, H. et al. Ultrathin Nanosheet-Assembled Co-Fe Hydroxide Nanotubes: Sacrificial Template Synthesis, Topotactic Transformation, and Their Application as Electrocatalysts for Efficient Oxygen Evolution Reaction. *ACS Appl. Mater. Interfaces* **12**, 46578-46587, (2020).

22 Maheskumar, V., Saravanakumar, K., Govindan, J., Park, C. M. Rational design of double perovskite La_2_Ni_0.5_Co_0.5_MnO_6_ decorated polyaniline array on MoO_3_ nanobelts with strong heterointerface boosting oxygen evolution reaction and urea oxidation. *Appl. Surf. Sci.* **612**, 155737, (2023).

23 Nai, J. W. et al. Construction of Ni(CN)_2_/NiSe_2_ Heterostructures by Stepwise Topochemical Pathways for Efficient Electrocatalytic Oxygen Evolution. *Adv. Mater.* **34**, 2104405, (2022).

24 Hu, J. et al. A universal electrochemical activation enabling lattice oxygen activation in nickel-based catalyst for efficient water oxidation. *Chem. Eng. J.* **430**, 132736, (2022).

25 Zhao, T. W. et al. Vertical Growth of Porous Perovskite Nanoarrays on Nickel Foam for Eﬃcient Oxygen Evolution Reaction. *ACS Sustainable Chem. Eng.* **8**, 4863-4870, (2020).

26 Selvadurai A, P. B. et al. Tailoring the cationic and anionic sites of LaFeO_3_-based perovskite generates multiple vacancies for efficient water oxidation. *J. Mater. Chem. A* **9**, 16906-16916, (2021).

27 Zhu, W. et al. Core-Shell Co-Co_x_ P Nanoparticle-Embedded N-Doped Carbon Nanowhiskers Hollow Sphere for Eﬃcient Oxygen Evolution Electrocatalysis. *Adv. Funct. Mater.* 2409390, (2024).

28 Kafle, A., Gupta, D., Bordoloi, A., Nagaiah, T. C. Self-standing Fe_3_O_4_ decorated paper electrode as a binder-free trifunctional electrode for electrochemical ammonia synthesis and Zn-O_2_ batteries. *Nanoscale* **14**, 16590-16601, (2022).
